# Genomic characterization of the world’s longest selection experiment in mouse reveals the complexity of polygenic traits

**DOI:** 10.1186/s12915-022-01248-9

**Published:** 2022-02-21

**Authors:** Sergio E. Palma-Vera, Henry Reyer, Martina Langhammer, Norbert Reinsch, Lorena Derezanin, Joerns Fickel, Saber Qanbari, Joachim M. Weitzel, Soeren Franzenburg, Georg Hemmrich-Stanisak, Jennifer Schoen

**Affiliations:** 1 Institute of Reproductive Biology, Research Institute for Farm Animal Biology (FBN), Dummerstorf, Germany; 2 Institute of Genome Biology, Research Institute for Farm Animal Biology (FBN), Dummerstorf, Germany; 3 Institute of Genetics and Biometry, Research Institute for Farm Animal Biology (FBN), Dummerstorf, Germany; 4 Department of Evolutionary Genetics, Research Institute for Zoo and Wildlife Research (IZW), Berlin, Germany; 5 University of Potsdam, Institute for Biochemistry and Biology, Potsdam, Germany; 6 Institute of Clinical Molecular Biology (IKMB), Kiel, Germany; 7 Department of Reproduction Biology, Research Institute for Zoo and Wildlife Research (IZW), Berlin, Germany

**Keywords:** Mouse, Fertility, Body mass, Endurance, Selective breeding, Genetic drift, Bottleneck, Whole genome sequencing, Single-nucleotide polymorphism, Structural variation

## Abstract

**Background:**

Long-term selection experiments are a powerful tool to understand the genetic background of complex traits. The longest of such experiments has been conducted in the Research Institute for Farm Animal Biology (FBN), generating extreme mouse lines with increased fertility, body mass, protein mass and endurance. For >140 generations, these lines have been maintained alongside an unselected control line, representing a valuable resource for understanding the genetic basis of polygenic traits. However, their history and genomes have not been reported in a comprehensive manner yet. Therefore, the aim of this study is to provide a summary of the breeding history and phenotypic traits of these lines along with their genomic characteristics. We further attempt to decipher the effects of the observed line-specific patterns of genetic variation on each of the selected traits.

**Results:**

Over the course of >140 generations, selection on the control line has given rise to two extremely fertile lines (>20 pups per litter each), two giant growth lines (one lean, one obese) and one long-distance running line. Whole genome sequencing analysis on 25 animals per line revealed line-specific patterns of genetic variation among lines, as well as high levels of homozygosity within lines. This high degree of distinctiveness results from the combined effects of long-term continuous selection, genetic drift, population bottleneck and isolation. Detection of line-specific patterns of genetic differentiation and structural variation revealed multiple candidate genes behind the improvement of the selected traits.

**Conclusions:**

The genomes of the Dummerstorf trait-selected mouse lines display distinct patterns of genomic variation harbouring multiple trait-relevant genes. Low levels of within-line genetic diversity indicate that many of the beneficial alleles have arrived to fixation alongside with neutral alleles. This study represents the first step in deciphering the influence of selection and neutral evolutionary forces on the genomes of these extreme mouse lines and depicts the genetic complexity underlying polygenic traits.

**Supplementary Information:**

The online version contains supplementary material available at 10.1186/s12915-022-01248-9.

## Background

Artificial selection is the selective breeding of organisms by which desired phenotypic traits evolve in a population [1]. Farm animals are the result of this selective breeding process to achieve efficient food production. However, artificial selection can also be applied experimentally in other species in order to connect genes and other genomic elements to selection response for complex traits such as behaviour [2] and limb elongation [3]. More generally, experimental evolution, which includes artificial selection experiments, is a powerful approach to understand response to selection across multiple traits and organisms [4].

The worldwide longest selection experiment on mice began in 1969 at the former Forschungszentrum für Tierproduktion (FZT), nowadays called Research Institute for Farm Animal Biology (FBN) located in Dummerstorf, Germany [5, 6]. Starting from a single founder line developed from four outbred and four inbred mouse strains [5, 6], selection lines for different complex traits were bred with population sizes of 60–100 breeding pairs per line. An unselected control line from the same founder line was maintained over the entire selection period with a larger population size (125–200 breeding pairs) [5, 6]. Over the course of >140 generations, selection has shaped the genomes of the Dummerstorf trait-selected mouse lines, and led to extreme phenotypes that include increased litter size (approx. double the litter size of the unselected mouse line) [7], body mass (approx. 90g body weight at 6 weeks of age) [8] and endurance (more than 3× higher untrained running capacity) [9, 10]. Therefore, in order to elucidate the unpredictable polygenic background of these complex traits, where multiple genes, regulatory elements and pathways act in conjunction, the Dummerstorf trait-selected mouse lines represent a valuable resource.

Other selection experiments have generated mice with increased litter size [11–14], as well as mice with enhanced body weight (see [15, 16] for a list of body weight mouse lines) and exercise performance [17], yet few studies have examined the polygenic background of these traits through genomic analysis. For example, a genome-wide association study of the high-fertility inbred strain QSi5 corroborated multiple previously reported loci associated with reproductive performance [18]. Likewise, a multi-line approach detected shared loci controlling body weight across seven high body weight selection lines, including an inbred subline of the Dummerstorf’s body mass line [16]. Finally, a comprehensive genomic analysis of mice from the “High Runner” selection experiment found widespread regions with significant genetic differentiation between selected and unselected replicate lines (4 per group) [19].

The Dummerstorf mouse lines expand the repertoire of polygenic mouse models to understand the genetic basis of fertility, body weight and endurance. Each of these lines arose from almost the same genetic diversity and has been maintained to this day for about half a century. Here we describe the selection history of this unique selection experiment, characterize line-specific patterns of genetic variation and identify genes that are likely associated to each selection trait.

## Results and discussion

### Phenotypic impact of selection

**Fig. 1:**
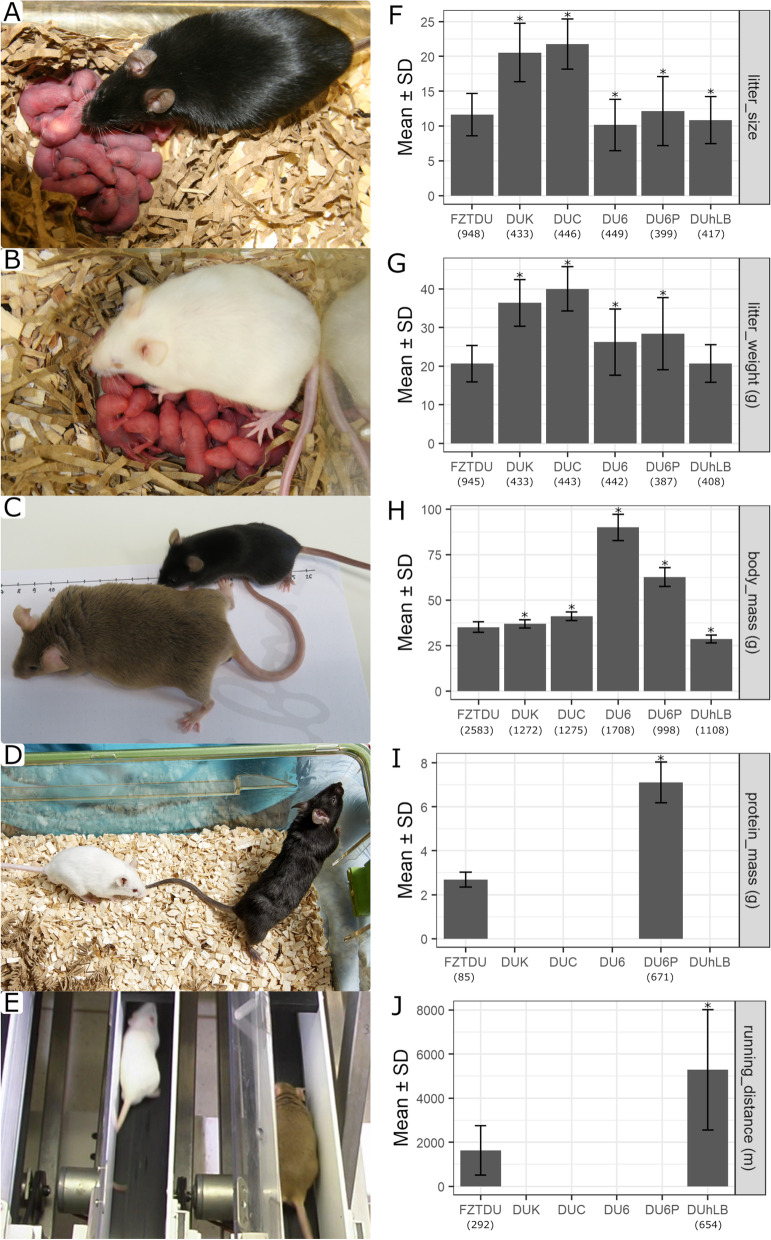
Phenotypic characteristics of the five trait-selected Dummerstorf mouse lines and the unselected control line FZTDU. Representative subjects showing the impressive litter size of DUK and DUC (**A**, **B**, **F**, **G**) and the considerable body size difference at 6 weeks of age between DU6 (**C**, **H**) or DU6P (**D**, **H**, **I**) and FZTDU. **E** Untrained mice undergoing a treadmill running endurance trial and the increased running performance of DUhLB due to selection (**J**). Stars signify differences (*p* < 0.05) after conducting a *t*-test between trait-selected lines and FZTDU. Sample sizes are indicated below tick labels (*x*-axis)

Over the course of more than 140 generations (Table 1), the selected traits (Table 2) have shown remarkable increments in each line (Table 1, Fig. 1, Additional file 2: Figure S1). The span and number of generations makes the present study the longest selection experiment ever reported in mice. Relative to the unselected control line FZTDU (exposed to genetic drift only), reproductive performance has doubled in DUK (Fertility mouse line 1) and DUC (Fertility mouse line 2) (Fig. 1A,B, F,G, Additional file 2: Figure S1). Even though these two trait-selected lines have achieved comparable litter sizes at first delivery (>20 offspring) [20], their reproductive lifespan differs, with 5.8 and 2.7 litters in average per lifetime for DUK and DUC, respectively [20]. A remarkable level of divergence has been achieved by the increased body size lines (Fig. 1C,D, Additional file 2: Figure S1). Individuals of the body mass line (DU6) have almost tripled their weight compared to FZTDU (Fig. 1H, Additional file 2: Figure S1), whereas mice of the protein mass line (DU6P) not only have become larger and heavier than FZTDU mice, but their level of muscularity is also considerably higher (Fig. 1D,I, Additional file 2: Figure S1). In terms of running distance capacity, the treadmill performance line (DUhLB) can on average cover three times more distance than FZTDU (Fig. 1J, Additional file 2: Figure S1).

**Table 1:** Summary selection history of the Dummerstorf mouse lines

	FZTDU	DUK	DUC	DU6	DU6P	DUhLB
Established (year)	1969	1971	1971	1975	1975	1982
No. Founders (BPs)	NR	60	60	80	80	100
Trait increment	–	2×	. 2×	3×	2×	3×
Percentage selected^a^	–	25–80	25–80	45–90	45–70	40–100
Relocation^b^ at generation	160–164	165	163–164	154–155	154–155	120–121
BPs per generation before relocation	200	60–100	60–100	60–80	60–80	60–100
BPs after relocation (founders)	55	19	24	7	19	22
BPs per generation (current)	125	60	60	60–120	60	60
End of selection (at generation)	–	Ongoing	Ongoing	Ongoing	152	141
No. generations under selection^c^	–	182/189	180/187	169/177	152/152	117/117
WGS at generation(s)	188/195	188/195	186/193	177/185	177/184	143/150
Alternative names^d^	Fzt: DU, DUK, Ctrl	DU-K, FL1	DU-C, FL2	BW, Titan	PA	DU-hTP

*BPs* breeding pairs, *WGS* whole genome sequencing, *NR* no records

Trait increment: mean trait expression in the sampled generation compared with trait expression in starting generation

^a^Percentage selected: percentage of litters from which parents were chosen

^b^Transfer of animals to a new housing building in 2011

^c^Total generations under selection until first and second sampling

^d^See Additional file 3: Table S1 for references on alternative names

**Table 2:** Selection criteria for Dummerstorf trait-selected mouse lines

Line-ID	Selected Sex	Trait
FZTDU	–	Unselected
DUK	Females	Number of offspring in first litter and litter weight at birth
DUC	Females	Number of offspring in first litter and litter weight at birth
DU6	Males	Body mass at day 42 of age
DU6P	Males	Protein amount in carcass at day 42 of age
DUhLB	Males	Submaximal untrained running distance on treadmill

With the exception of the obese line DU6 [21], each one of the trait-selected mouse lines has developed an extreme phenotype without obvious detrimental effects on their general health, well-being, and longevity. All these lines are still maintained, but selection only continues for DUK, DUC and DU6. Due to the long span of this selection experiment, lines have been given alternative names (Table 1, Additional file 3 [6, 8, 10, 20–41]: Table S1) and selected at variable intensities (Additional file 2: Figure S2).

### Whole genome sequencing (WGS) analysis and short variant detection

After quality filtering and trimming, >90% of the raw reads were mapped to the genome as pairs, with a mean insert size of ~380 bp. For samples sequenced at a target coverage of 30×, mean genome-wide coverage averaged ~24×, with ~95% of genome territory covered at least 5×; samples sequenced at a target coverage of 5× averaged ~8× and ~72%, respectively (for a summary across all samples see Table 3 and for details, Additional file 4: Data S1).

**Table 3:** Summary metrics WGS data

	Target coverage 30×	Target coverage 5×
Sample size	60	90
Mean number of reads mapped as pairs	90.72%	93.07%
Mean insert size	347.73 bp	401.65 bp
Mean genome-wide coverage	24.08×	7.89×
Mean genome territory covered ≥ 5×	95.57%	71.82%

**Fig. 2:**
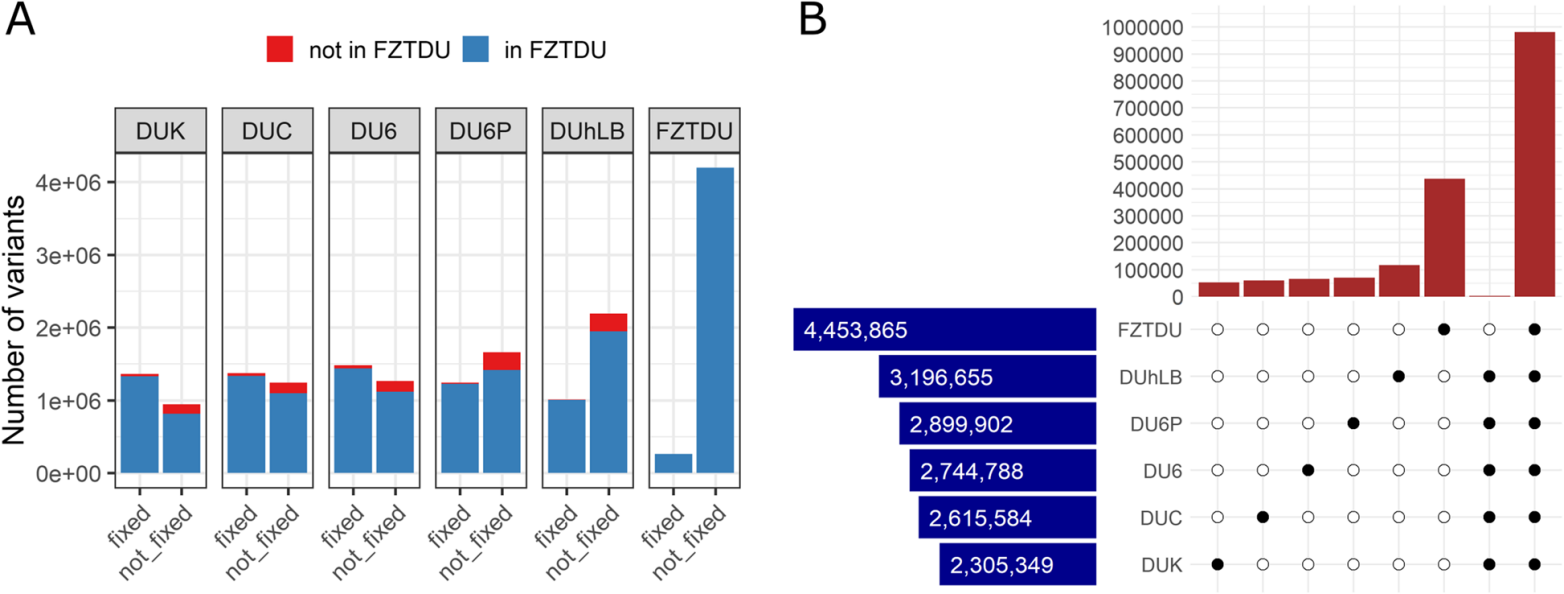
Overview and classification of SNP sites. **A** SNP sites were classified as fixed or not-fixed if their allele frequencies were 1 or <1, respectively. At each line, the fraction of variants shared (in FZTDU) and not shared (not in FZTDU) with FZTDU is also shown. **B** Blue horizontal bars: Total number of SNP sites detected in each line. Brown vertical bars: Number of private SNP sites for each line (single black dots), shared only by the trait-selected lines (five black dots) and by all lines (six black dots)

The final variant call set contained 5,099,945 single-nucleotide polymorphisms (SNPs) and 766,655 insertions-deletions (INDELs) (374,604 insertions; 392,051 deletions, Additional file 2: Figure S3B). The trait-selected lines had much fewer variants than FZTDU and these variants were mostly fixed, whereas FZTDU variants were mostly polymorphic (Fig. 2, Additional file 2: Figure S4, Additional file 3: Table S2). This reduction in genetic diversity could be explained by the fact that the trait-selected lines have been maintained at smaller population sizes and were relocated with fewer founders (Table 1). In fact, it has been shown that artificial selection for complex traits does not affect the number of segregating sites [3], nor the number of SNP sites and heterozygosity [19]. Interestingly, more than 89% of the variants observed in the trait-selected lines were also detected in the control line FZTDU (Fig. 2A, Additional file 3: Table S2), indicating that despite genetic drift, the control line preserves most of the alleles underlying each selected trait and that it still is a proxy of the original founder population.

Most (~90%) INDELs were no longer than 10 bp (Additional file 2: Figure S3A, Additional File 3: Table S4), with slightly more deletions than inversions (Additional file 2: Figure S3B). The proportion of SNPs and INDELs overlapping dbSNP was 95% and 55%, respectively. This discrepancy is not necessarily due to a high number of artefacts in the INDEL set, but rather by the fact that INDELs are a much less characterized type of genetic variant in comparison [42].

The number of alleles present in all six lines was ~1M, but very few alleles were shared by the trait-selected lines exclusively (~3.3K) (Fig. 2B). The lines DU6P and DUhLB were the most polymorphic of the trait-selected lines, followed by DU6. The two fertility lines (DUK, DUC) were the least polymorphic ones (Fig. 2B, Additional file 3: Table S2).

Almost all SNPs and INDELs (~97%) occurred in non-coding regions (introns ~56%; intergenic ~41%). This is not an unexpected outcome considering that only ~2% of the genome codes for proteins and genetic variation is widespread. Inter-genic variants could affect regulatory elements of gene expression, as well as transcripts not yet described [43], whereas intronic variants could affect gene splicing [44].

Based on assessment of variant annotations, a very small number of variants (20,236 SNPs and 1,801 INDELs) were classified as high-impact and moderate-impact mutations, and could interfere with gene transcription or translation. These “impact variants” were screened for (i) being private for any trait-selected line (Additional file 3: Table S3) and (ii) the functional categories their affected genes belonged to. The number of genes affected by these private “impact variants” was twice as large in DUhLB (1027 genes) than in the other trait-selected lines (465–546 genes). However, there was no obvious coherence between significantly enriched functions and the selected traits (Additional file 4: Data S2).

### Runs of homozygosity (RoH) and linkage disequilibrium (LD)

**Fig. 3:**
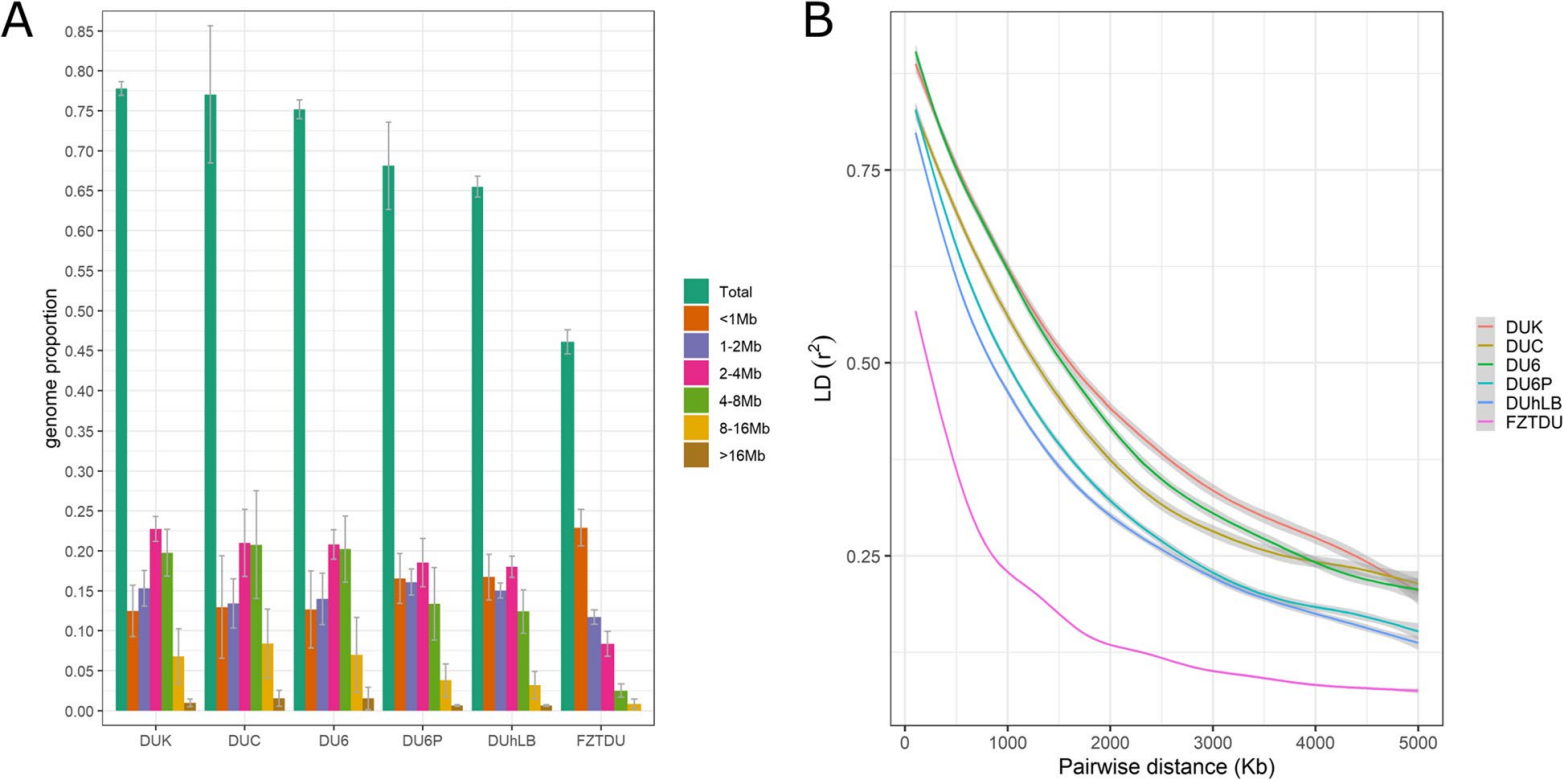
Runs of homozygosity and linkage disequilibrium decay in the Dummerstorf mouse lines. **A** Per line average extent of homozygosity as a fraction of the genome length. RoH of different length range are specified by colours. Error bars show ±1SD. **B** Decay of the mean genotype correlations among SNP pairs as close as 0.1 Mb and as far as 5 Mb

While for the five trait-selected lines, most of the SNP loci (57.5–81.5%) were already fixed for either the reference or the alternative allele, in the control line FZTDU alleles were mostly (>75%) polymorphic (Additional file 2: Figure S5). This disparity was also reflected by the distribution of frequencies for the alternative allele, displaying a “U” shape that is much more pronounced in the trait-selected lines than in the control line (Additional file 2: Figure S6). Genomes of mice from the control line FZTDU also had higher nucleotide diversity (Additional file 2: Figure S7 and S8). Accordingly, RoH covered between ~65 and ~78% (~50% as 1–8 Mb tracts) of the genome length of the trait-selected lines, but only ~45% (~23% as 1–8 Mb tracts) of the genome length of FZTDU (Fig. 3A). Analysing RoH shared among individuals of a population can aid to detect past selection events [45]; however, this is applicable as long as RoH events are rare in the genome (RoH islands), which is not the case here, where RoH are widespread, indicating that the observed degree of homozygosity is the result of a combination of multiple evolutionary forces.

Linkage disequilibrium decay, represented by the genotype correlation (*r*^2^﻿) between pairs of SNP sites within min. 0.1 Mb and max. 5 Mb, can be classified into three patterns with decreasing decay strength; one for the three most homozygous trait-selected lines (DUK, DUC and DU6; upper three lines Fig. 3B), a second for the two least homozygous trait-selected lines (DU6P and DUhLB; middle two lines Fig. 3B) and a third for the unselected line FZTDU (bottom line Fig. 3B). Overall, *r*^2^ clearly differs between trait-selected lines and FZTDU. Comparable levels of *r*^2^ have been reported in mountain gorillas, in which population decline has led to high levels of inbreeding [46]. Likewise, strong levels of LD have been observed in laboratory mice [47]. However, other populations with high levels of inbreeding, such as dog [48] and horse breeds [49], do not display such strong genotypic correlations, highlighting the impact of the bottleneck in the genetic diversity of the Dummerstorf mouse lines.

### Population structure of the Dummerstorf mouse lines

**Fig. 4:**
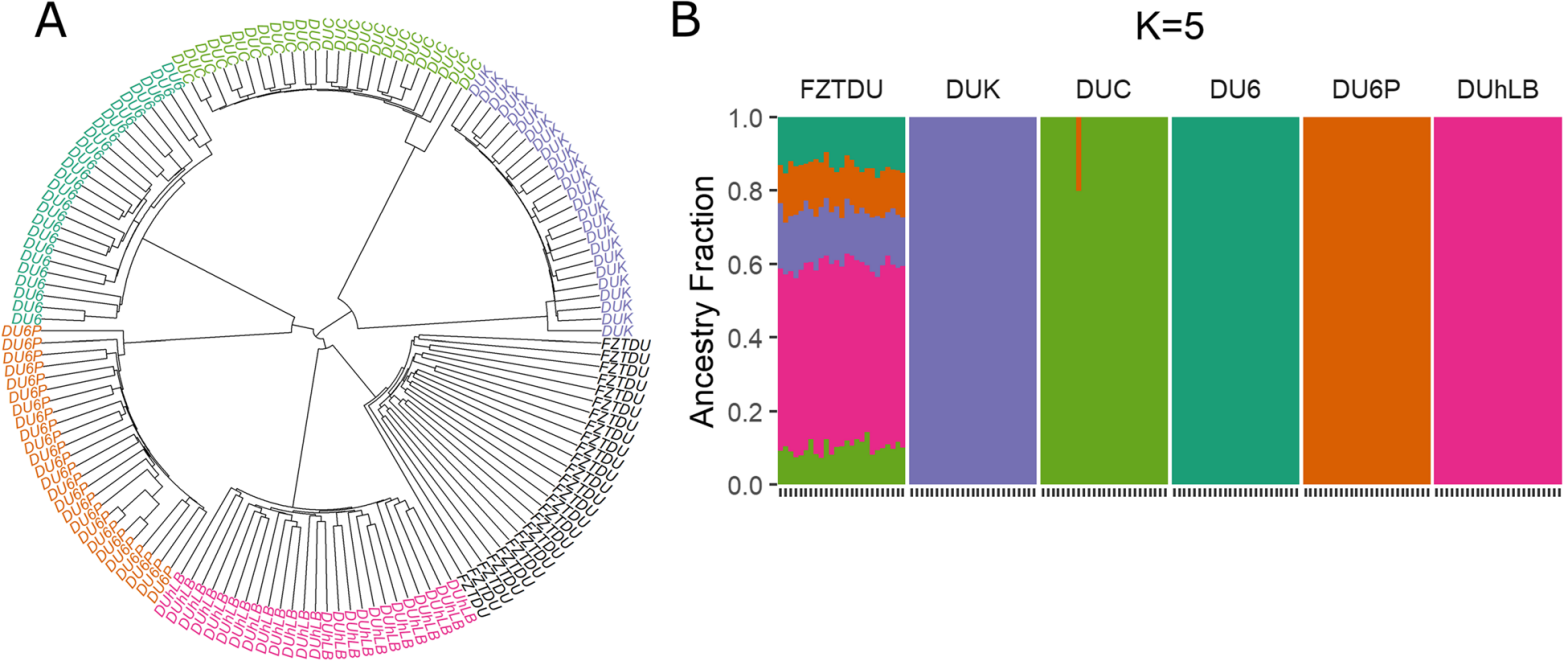
Genetic structure and cluster assignment of 150 mice of the six Dummerstorf mouse lines. **A** Hierarchical clustering analysis assorting individuals into distinctive mouse line clusters. **B** Genetic composition of each mouse (indicated by 25 ticks on the *x*-axis) in terms of the five trait-selected lines. Individuals are coloured according to the respective line of origin

The genetic relationship among the 150 Dummerstorf mice was assessed by hierarchical clustering (HC) and admixture analysis using the 5,099,945 SNPs obtained after variant calling. Samples formed a hierarchical group structure that represented each of the Dummerstorf lines (Fig. 4A). There was no admixture present in the trait-selected lines, except for one DUC animal sharing ancestry with mice from DU6P (Fig. 4B). FZTDU is represented as an admixture of all the trait-selected lines with similarly large contributions of the four older lines and a significantly larger contribution of DUhLB (Fig. 4B). This is expected because this mouse line is the youngest and has had the least number of generations that underwent selection.

### Genetic differentiation of the trait-selected lines

**Fig. 5:**
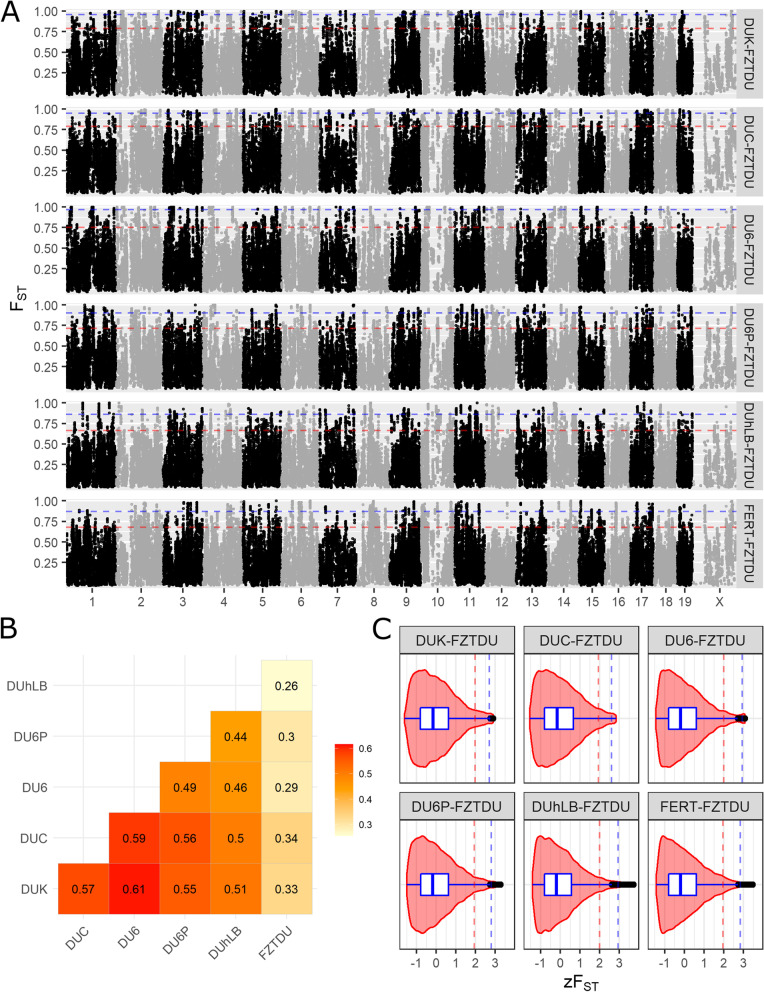
Genetic differentiation of the Dummerstorf trait-selected lines. **A** Genome-wide scans of genetic differentiation in sliding window mode (size = 50 kb, step = 25 kb) contrasting each trait-selected line to FZTDU. Each window is the average *F*_ST_ of at least 10 SNPs. **B** Pairwise genomic mean *F*_ST_ among all six Dummerstorf lines. **C** *F*_ST_ distribution as *z*-scores, illustrating the departure of each window from the mean genomic level of genetic differentiation. Dotted lines indicate the 95th (red) and 99th (blue) percentiles and black dots correspond to data points larger than 1.5 the interquartile range (outliers)

Mean genome-wide pairwise genetic differentiation among trait-selected lines estimated with the genetic differentiation index (*F*_ST_) ranged from 0.44 to 0.61 (Fig. 5B). The highest level of differentiation was found between either one of the fertility lines and the body mass line DU6 (*F*_ST(DUK-DU6)_ = 0.61 and *F*_ST(DUC-DU6)_ = 0.59; Fig. 5B), followed by the differentiation between the two fertility lines themselves (*F*_ST(DUK-DUC)_ = 0.57; Fig. 5B). Although pairwise genetic differentiation between trait-selected lines and the control line was similar in all comparisons (*F*_ST_ ~ 0.3), it was lowest in the pairwise comparison between the two most polymorphic lines (*F*_ST(DUhLB-FZTDU)_ = 0.26; Fig. 5B). Such strong levels of differentiation occur mainly as a result of reproductive isolation and genetic drift [50]; however, it is expected that a subset of alleles that have arrived to fixation due to selection contribute to genetic differentiation as well. The challenge is thus to sort out which genomic regions contain such beneficial alleles.

### Trait-specific regions of genetic differentiation

**Fig. 6:**
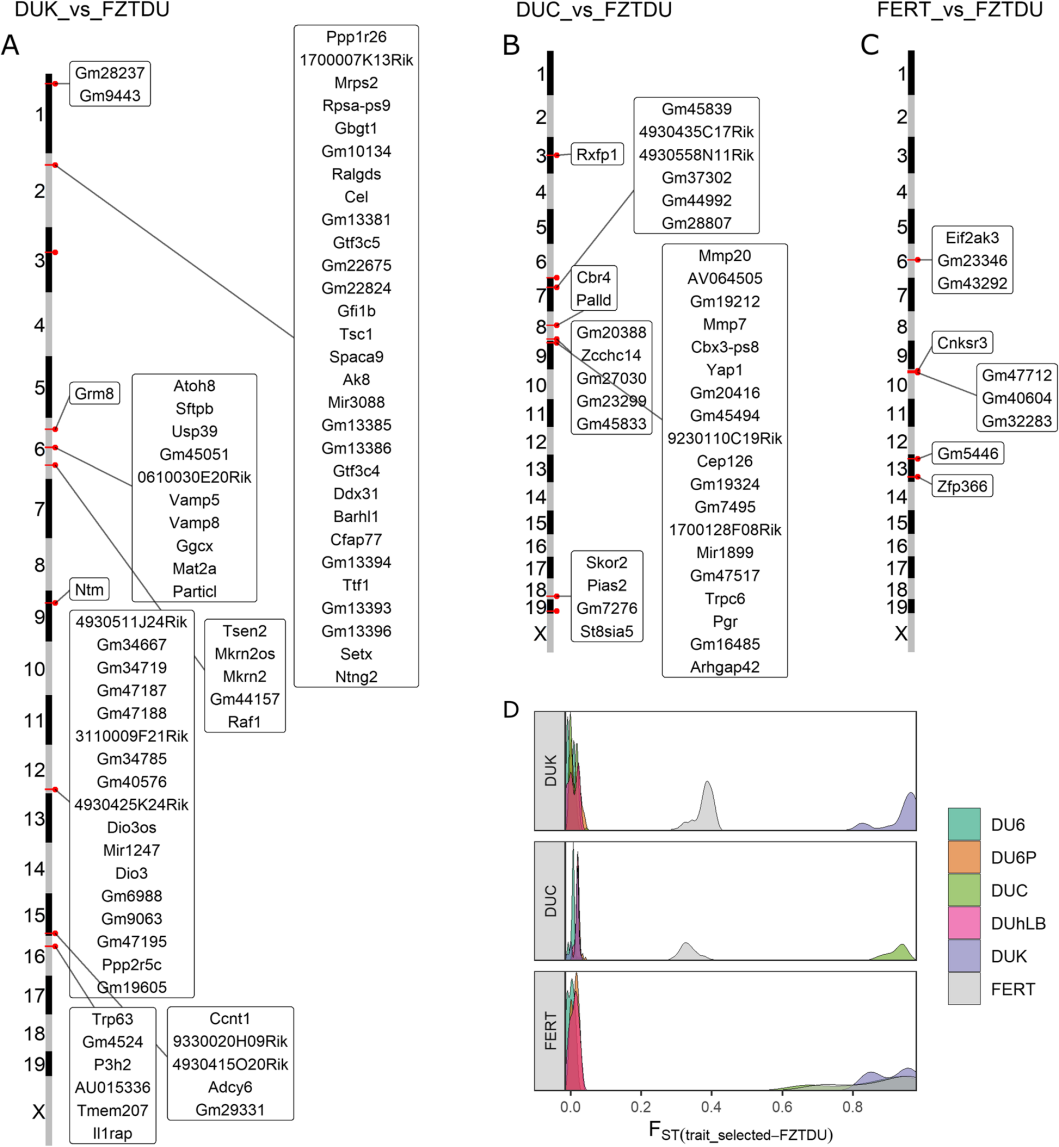
Genes mapped to regions of distinct genetic differentiation for fertility lines. **A–C** Genomic overview of RDDs for each of the fertility lines (DUK, DUC) and the joint pseudo-line (FERT). **D** *F*_ST_ distribution of RDDs, demonstrating the gap in *F*_ST_ between the target lines and the rest

**Fig. 7:**
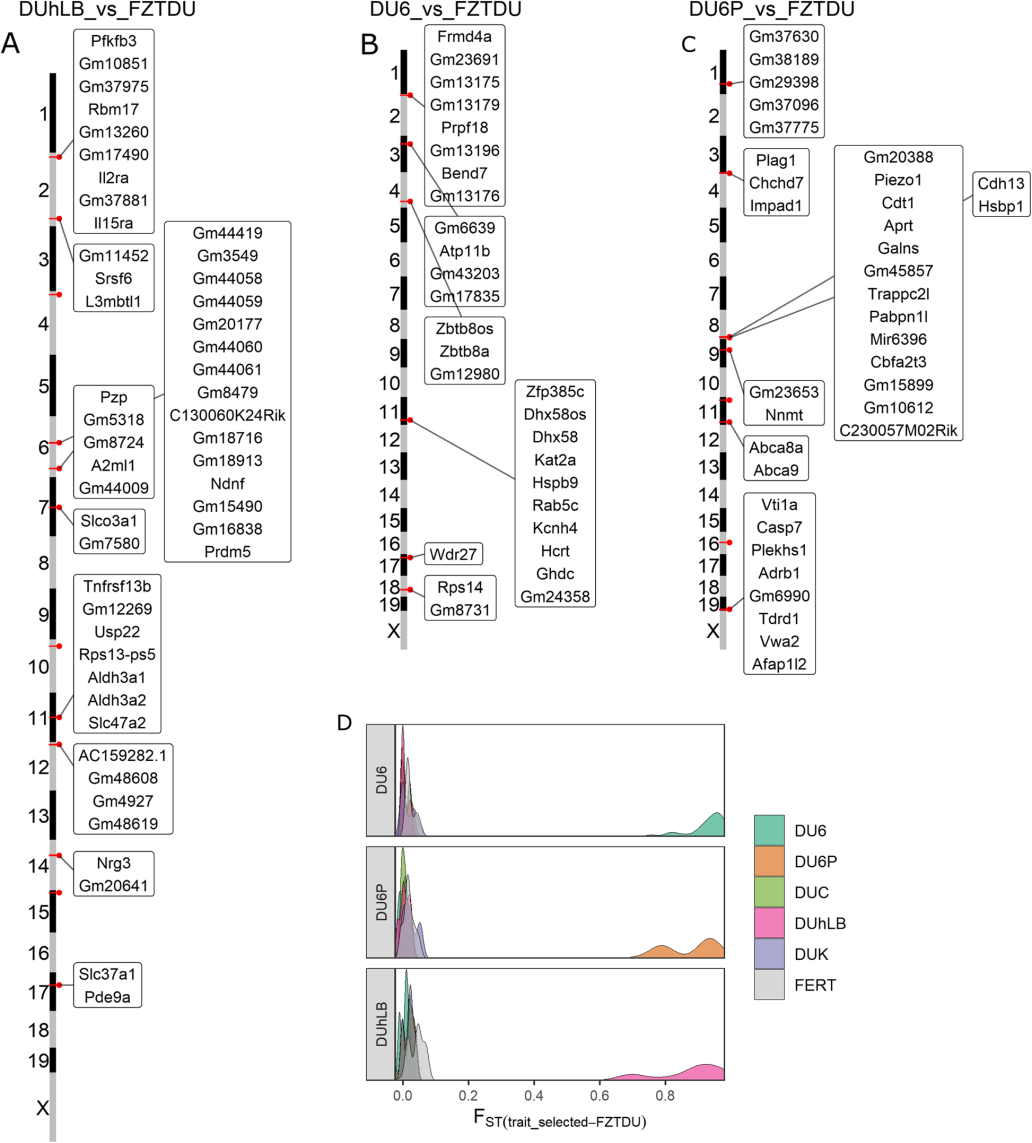
Genes mapped to regions of distinct genetic differentiation for body mass and treadmill performance lines. **A–C** Genomic overview of RDDs for DUhLB, DU6 and DU6P, respectively. **D** *F*_ST_ distribution of RDDs, demonstrating the gap in *F*_ST_ between the target lines and the rest

Genome-wide scans were conducted in order to detect genomic regions of consistent genetic differentiation between each trait-selected line and FZTDU. The pseudo-line of DUK and DUC combined (FERT) was also included, for a total of six *F*_ST_ contrasts. Overall, outstanding regions of particularly extreme genetic differentiation were not observed, but rather a uniform genome-wide level of high *F*_ST_ (Fig. 5A). Choosing genomic regions of interest by focusing on the most differentiated regions (95th or 99th percentile of the *F*_ST_ distribution) resulted in the detection of multiple loci in every chromosome (Fig. 5A). Because these regions were frequent and did not sufficiently depart from the global level of genetic differentiation to be considered genomic outliers (i.e. max. zF_ST_: 2.89–3.47, Fig. 5C), a more stringent approach was applied to identify line-specific regions of high genetic differentiation (Fig. 6D and Fig. 7D), while reducing the influence of genetic drift. These regions of distinct genetic differentiation (hereafter referred to as RDDs) appeared simultaneously in the top 5% *F*_ST_ windows of the target contrast and in the bottom 10% of all the remaining contrasts, occurring close to each other in only a subset of chromosomes and containing multiple genes (Fig. 6A–C, Fig. 7A–C, Additional file 4: Data S3-S14), some of which were related to the selected traits (see below).

These thresholds were empirically determined based on a similar study comparing two extremely differentiated inbred maize lines [51]. Neutrality simulations were not conducted due to the lack of genetic material from founders and incomplete pedigrees. This information is critical to identify discrete candidate targets of selection for complex traits, in which selection response occurs gradually and myriads of loci with small effects are expected to be involved [3].

### Line-specific patterns of structural variation (SV)

Despite primarily thought to be deleterious and implicated in disease phenotypes [52, 53], large chromosomal rearrangements such as deletions, duplications and inversions have an important role in local adaptation and divergence of populations [54]. These structural variants can lead to gene expression differences by disrupting genes and altering gene dosage [55]. Because copy number variation often results in notable phenotypic differences, it is likely a subject to selection during domestication [56]. For example, genes related to metabolic activity and production traits have been shown to be affected by copy number variation during artificial selection of cattle [57], goats [58] and pigs [59].

After calling and filtering, only duplications, deletions and inversions remained in the final SV data set. Insertions did not occur in enough samples to be included in the analysis. Also, because of the lower detectability in the low sequencing coverage samples, most SVs were found in high coverage samples (Additional file 3: Table S10). Nevertheless, the final SV call set contained the union of good-quality SVs detected in both coverage sets.

SVs were predominantly located in non-coding regions (98%) where they could affect gene expression. Also, SVs (Table 4) were more abundant in the trait-selected lines (deletions (DEL) 5560–4339; duplications (DUP) 48–20; inversions (INV) 1508–530) than in the control line (DEL 3902; DUP 14, INV 605) implying that large genomic rearrangements could contribute to the development of the selected traits. In order to associate SVs to each selected trait, line-specific SVs overlapping protein-coding genes were identified and characterized in greater detail (Additional file 4: Data S15). The total number of these line-specific SVs ranged from 9 (FZTDU) to 36 (DUC), comprising mostly deletions and inversions (Table 4). Most SVs were polymorphic and large length differences were observed between polymorphic and fixed SVs (Additional file 3: Table S6). Fixed line-specific deletions were detected in all lines, whereas duplications were found only in DU6P, and inversions only in DUC, DU6P and DUhLB (Additional file 3: Table S7).

**Table 4:** Summary of structural variants detected in all mouse lines

	Total				Line-specific-genic			
	DEL	DUP	INV	Total	DEL	DUP	INV	Total
DUK	4633	32	530	5195	11	2	7	20
DUC	5560	48	1248	6856	10	2	24	36
DU6	5025	27	551	5603	11	0	7	18
DU6P	4339	23	2091	6453	9	1	14	24
DUhLB	4614	20	1508	6142	10	1	9	20
FZTDU	3902	14	605	4521	4	0	5	9

The number of genes affected by fixed line-specific SVs varied from 1 (DUC, DU6P, FZTDU) to 5 (DUK), but went up to more than a thousand for genes affected by large polymorphic inversions (Additional file 3: Table S8). These genes were classified in functional groups based on the biological processes they are associated with (Additional file 3: Table S9). The most gene-rich functional groups are the ones associated with sensory perception, predominantly olfaction (found in the fertility lines DUK and DUC), followed by “cell cycle and nucleic acid transcription and translation” (in DUC), and “metabolism and energy conversion” (DUC, DU6P).

### Genes associated with fertility

Genes detected in RDDs for DUK were enriched for “phospholipase D signalling pathway” (Additional file 3: Table S5). In granulosa cells, phospholipase D activity is stimulated by GnRH, thereby inducing or inhibiting cell differentiation depending on the maturation state of the ovarian follicle [60]. Other genes encode for proteins involved in the ovarian development and maintenance of the primordial follicle reserve (*Tsc1* [61], *Trp63* [62]), in the vascularization of the placenta (*Atoh8* [63]) and facilitate maternal supplied lipids and dietary fat digestion in neonatal mice (*Cel* [64, 65]). Furthermore, DUK shares a fecundity associated region (*Sftpb*, *Usp39*, *Tmem150*, *Rnf181*, *Vamp5*, *Vamp8*, *Cgcx*, *Mat2a*) with Qsi5 mice [18], an inbred mouse line known for its increased litter size, and candidate genes associated with birth rate and male fertility in humans (*Ntm* [66]) and litter size in cattle, goats and pigs (*Dio3* [67–69]). Interestingly, analysis of private SVs detected a 317-bp deletion affecting *Olfr279* (Additional file 4: Data S15). This gene has been associated to mouse male sub-fertility [70] and more generally, olfactory receptors could regulate fertilization [71, 72].

Significantly enriched terms for DUC included “intracellular steroid hormone receptor signalling pathway” (Additional file 3: Table S5), involving progesterone receptor (*Pgr*) carrying a missense mutation, which is fixed in and specific for DUC (Additional file 2: Figure S9B). Progesterone is one of the main steroid hormones regulating reproductive processes and critical for (i.a.) pregnancy maintenance and mammary gland development [73, 74]. It remains to be proven if a connection exists between this missense and potentially deleterious (Sorting Intolerant From Tolerant (SIFT) score = 0.04) mutation and the fact that DUC females display increased levels of progesterone [22]. Interestingly, a Neanderthal missense mutation in *Pgr* associated with increased fertility was recently reported to segregate in human populations [75]. Further candidates in DUC control ovarian follicle development, uterine growth and endometrial angiogenesis during pregnancy (*Yap1* [76], *Rxfp1* [77, 78]). In the context of preparation of the endometrium for implantation and pregnancy and progesterone signalling, the gene *Rrm2* [78] was identified by the structural variation analysis of the DUC genome.

The fertility lines DUK and DUC have been bred according to the same criteria, share the same evolutionary history, and both have been able to more than double the number of pups per litter since the beginning of selection. Despite these commonalities, improved fertility is achieved via different physiological pathways in each line [22]. For example, females from both fertility lines have an increased ovulation rate, but only DUK exhibits follicles containing multiple oocytes; DUC on the other hand shows an increased progesterone level compared to DUK and FZTDU [22]. The scarce number of RDDs in the combined FERT population also illustrates this discrepancy. Candidate RDD and line-specific SV overlapping genes in both fertility lines likely affect the reproductive process on multiple levels such as ovarian physiology, placentation, sex steroid signalling and milk composition.

### Genes associated with body size and endurance

Two of the Dummerstorf trait-selected mouse lines have increased their body weight in response to selection. The “giant” DU6 line (selected for body mass at 6 weeks of age) exhibits an obese phenotype [8] while the protein-mass line DU6P (selected for protein mass in the carcass) is lean and muscular [25].

In line with the obese phenotype, DU6 candidate genes overlapping RDDs regulate energy metabolism and food intake (*Hcrt* [79]) and are linked to feed efficiency (*Wdr27* [80]) and body composition in other species (*Atp11b* [81]). DU6 mice also exhibit larger bones [21], and the analysis of SVs detected *Smad5*, a modulator of bone formation [82], to be partially overlapped by a heterozygous deletion and a heterozygous inversion. Though DU6 gave origin to DUHi, one of the lines used to detect parallel selected regions (PSRs) for high body weight, none of the RDDs intersected with PSRs [16]. This is partly explained by the fact that DUHi was established after sampling DU6 mice on generation 85 (well before bottleneck, see Table 1) and further maintenance of these animals under inbreeding [15].

Candidate genes in the RDDs for DU6P conform with growth-related major quantitative trait loci found in sheep and are known to influence stature and body size in cattle, pigs and human (*Plag1* [83, 84], *Chchd7* [83–85], *Impad1* [86]). In line with this, an SV (deletion) was found overlapping *Fam92a*, a gene that is involved in limb development [87]. Further candidates for lean body mass are the RDD overlapping genes *Piezo1* (myotube formation [88, 89]) and *Cdh13* (control of lipid content in developing adipocytes [90–92]).

Finally, genes specific for the endurance line DUhLB participate in lipid metabolism (these animals display faster mobilization of lipids during exercise). Only two DUhLB genes (*Aldh3a1* and *Aldh3a2*, the later containing 3 missense SNPs (Additional file 2: Figure S10C)) caused the significant enrichment of the “Histidine metabolism” and “beta-Alanine metabolism” pathways (Additional file 3: Table S5). The “marathon mice” DUhLB have developed a striking metabolic phenotype characterized by accelerated browning of subcutaneous fat and altered mitochondrial biogenesis in response to selection for high treadmill performance [29]. Likewise, detected RDD candidate genes are involved in the development of brown adipocytes (*Srsf6* [93]), removal of toxic waste products from lipid metabolism (*Aldh3a2* [94]), mobilization of fatty acids, mitochondria content and cristae complexity (*Il15r* [95]) and in the regulation of glycolysis associated to obesity and weight gain (*Pfkfb3* [96, 97]). Moreover, SV analysis detected a ~2.8 kb inversion in *Atp5j* whose overexpression has been shown to counteract exercise-induced cardiac hypertrophy in mice [98]. Interestingly, the genes identified here did not overlap with significantly differentiated genes of the “High Runner” selection experiment [19], highlighting the fact that these two studies produced phenotypically different mice (i.e. DUhLB shows lower running wheel activity compared to controls [31]).

### Limitations

There are five main weaknesses in this study. First, due to gaps in pedigree documentation over more than 140 generations, modelling neutrality was not feasible. In turn, the thresholds to evaluate line-specific genetic differentiation were chosen empirically by setting conservative limits that minimize the presence of false positives.

Second, at its origin in 1969, the study was not designed to conduct genomic analyses. Thus, genetic material from the founders is not available. Unfortunately, this and the incomplete pedigree information hamper the detection of signatures of selection. However, the genomic data generated here still allows deriving biological interpretations based on the line-specific patterns of genetic differentiation, which is the subject of this study.

Third, relocation of the mouse lines by embryo transfer resulted in a genetic bottleneck and random fixation events. This further obscures insight into the selection response mechanisms of these mouse lines. Still, the current strong phenotypic divergence of the lines is the result of long-term selection.

Fourth, except for the fertility lines DUK and DUC, trait-selected lines were not replicated in order to identify overlapping genomic signatures. Interestingly, these two lines are markedly different both physiologically and genetically, despite having the same selection criteria.

Finally, SVs were detected using short pair-end reads (150bp) and this is not an optimal approach for SV discovery. For this, long reads provide much greater accuracy and sensitivity [99, 100].

## Conclusions

The genomes of the Dummerstorf trait-selected mouse lines have evolved in response to selective breeding and neutral forces, exhibit low genetic diversity and display distinct patterns of genetic variation. Distinguishing between selection and neutral evolution is a challenging task and will require further research. However, by focusing on regions of distinct genetic differentiation, we were able to identify genes with important functions associated to the selected traits.

Over the span of this selection experiment, traits have improved continuously and have not decayed despite the dramatic loss of genetic diversity within lines. This implies that many of the alleles that contribute to trait improvement have arrived to fixation and that these lines are highly enriched for such alleles. Therefore, a deeper understanding of the genomes of the trait-selected Dummerstorf mouse lines will provide valuable insights into the genetic basis of important polygenic traits and constitutes an unprecedented scientific resource for geneticists, physiologists and the wider biomedical research community.

## Methods

### Selection history of the Dummerstorf trait-selected mouse lines

The selection experiment started in 1969 (Tables 1 and 2, for more detail see Additional file 1: Supplementary Methods [5, 6, 22, 101–114]) with the establishment of a founder line FZTDU (Forschungszentrum für Tierproduktion Dummerstorf) [5, 6] by systematic crossing of four outbred strains (NMRI orig., Han:NMRI, CFW, CF1) and four inbred strains (CBA/Bln, AB/Bln, C57BL/Bln, XVII/Bln). From FZTDU, five lines were established through selective breeding: two lines were selected for increased litter size (DUK and DUC), one for increased body mass (DU6), and one each for protein mass (DU6P) and treadmill running endurance (DUhLB) (Table 2, Fig. 1, Additional file 2: Fig. S1).

### Sample collection and whole genome sequencing

All animal procedures were performed in accordance with national and international guidelines and approved by the Animal Protection Board of the Institute for Farm Animal Biology. Genomic DNA was purified from tail biopsy samples using QIAamp DNA Mini Kit (Qiagen, Hilden, Germany) according to the manufacturer’s recommendations. A total of 25 females per line (150 animals in total) were sampled at two different time-points (Table 1). For the first time-point, 10 females per line with the lowest kinship coefficient were chosen. Kinship was determined using the programme INBREED implemented in the software SAS/STAT® (v9.4, SAS Institute Inc., USA). For the second time-point, 15 females per line were chosen at random since the kinship coefficient is similar among subjects of the same line. The study was originally designed with 10 females per line, sequenced at high coverage (target 30×, time-point 1) to capture as much line-specific genetic variability as possible. Due to the low genetic variability in each line resulting from the preliminary data analysis, 15 additional females per line were sequenced. As this was intended to verify the low degree of genetic variability at the initially detected loci and to increase the number of total observations for each line, the samples of the second batch were sequenced with a lower sample coverage (target: 5×, time-point 2).

Library preparation and sequencing were carried out at the Competence Centre for Genomic Analysis (Kiel). Paired-end sequencing libraries were prepared using the TruSeq Nano DNA Library Prep kit following the manufacturer’s specifications (Illumina Inc., San Diego, CA, USA). Out of the 150 libraries, 60 were sequenced on a HiSeq 4000 platform (Illumina Inc.), and 90 samples were sequenced on a NovaSeq 6000 (Illumina Inc.) platform. The target coverage was 30× (high coverage set) and 5× (low coverage set), respectively. Read length was 151 nucleotides. Samples sequenced at 30× (*n* = 60) were distributed in 9 lanes for a total of 540 pairs of read files. Ten of those samples had to be supplemented with extra sequencing data due to not reaching the expected 30× coverage. Samples sequenced at 5× were not lane-distributed, amounting to 90 pairs of read files. In total, 640 pairs of read files were produced. Sample-wise WGS data is summarized in Additional file 4: Data S1.

### Analysis of WGS data

Adapter removal and quality trimming were done using Trimmomatic v0.38 [115] for HisSeq reads and FASTP v0.19.6 [116] for NovaSeq reads. Read quality was evaluated before and after processing with FastQC v0.11.5 [117]. Reads were aligned to the mouse genome build GRCm38.p6 [118, 119] from Ensembl version 93 [120] using the Burrow-Wheeler Aligner software in MEM mode (BWA-MEM) [121] coupled with SAMtools v1.5 [122] in order to store alignments as Binary Alignment Map (BAM) files. Per sample BAM files were processed sequentially with Picard tools [123] by adding read group information (*AddOrReplaceReadGroups*), merging alignments from different read groups (*MergeSamFiles*), and by sorting (*SortSam*) and marking duplicated (*MarkDuplicates*) reads.

### Short variant calling and annotation

Short variants were detected according to GATK’s best practices for germline short variant discovery (GATK v 4.0.6.0) [124–127]. Systematic errors in base quality were corrected using *BaseRecalibrator* and dbSNP [128] version 150 for *Mus musculus* (Ensembl version 93 [129]). For each sample, variants were called with *HaplotypeCaller* and then combined with *GenomicsDBImport*. Joint genotyping was done with *GenotypeGVCFs* and then only bi-allelic variants (SNPs and INDELs) were retained. Filtering was applied separately for SNPs and INDELs. Site-level filtering was done following the Variant Quality Score Recalibration (VQSR) procedure. This comprised an internal variant set used as truth-training resource, created after stringent site-level filtering of the bi-allelic variants obtained from joint genotype calling, plus an external pre-filtered training variant set provided by the Mouse Genomes Project (MGP version 5 [130]). Variants were genotyped as missing if the depth of coverage (DP) was either too low (<4), too high (3 standard deviations higher than the sample mean depth) or if the genotype quality (GQ) was too low (<20). INDELs overlapping microsatellites [131] were excluded. The final set consisted of variants present in at least 15 samples per line (except for DU6 that had a lower coverage, so this threshold was lowered to 12 samples). Annotations were done using SnpEff v4.3t [132] and missense mutations were further evaluated with Ensembl Variant Effect Predictor (VEP) v.101.0 [103] to obtain their corresponding SIFT scores [133] and to predict amino acid changes affecting protein function.

### Structural variant calling and annotation

Processed BAM files used for short variant calling were also used to detect large structural variants (SVs). SVs correspond to deletions, duplications, insertions, inversions and translocations of at least 50 bp in size [134]. Because of the considerable difference in coverage of the two sequence data sets, this was done independently for the high and the low coverage set.

Three SV callers (Manta v.1.6.0 [110], Whamg v.1.7.0 [111] and Lumpy v.0.2.13 [112]) were applied per line and per coverage set yielding six call sets per line (see Additional file 1 for more detailed information). Specific filters were applied depending upon the call set. SVs detected by Manta were site-filtered by excluding SVs with poor mapping quality (Mapping Quality (MAPQ) < 30) or with excessive coverage (>3 × the median chromosome depth) that could be due to reads originated from low complexity regions. For each sample, only SVs with GQ ≥ 20 and read depth ≥5× were accepted. Whamg SV calls with sizes <50 bp and >2 Mb were filtered out to improve call accuracy. Here too, only calls with read depth ≥ 5× were accepted. Calls with GQ < 20 were filtered out. To reduce the number of false positive calls, high cross-chromosomal mappings were excluded, as Whamg is aware of but does not specifically call translocations. Likewise, SVs in poorly mapped regions were also removed. Lumpy SV calls for which supporting evidence (FORMAT/SU field) was below 5 (SU<5) were excluded, as well as SV calls with GQ<20. Since both Whamg and Lumpy do not have a built-in genotyping module, SV call sets were genotyped with Svtyper v0.7.1 [101] prior filtering for genotype quality. For each line and coverage set, SVs called by at least two SV callers were merged using Survivor v.1.0.7 [102] and kept if they were found in at least 10 samples. The final set consisted of the union of SVs detected in the high and low coverage read sets. We then intersected SV calls among all six mouse lines to obtain SVs private for each line (line-specific) and SVs shared among lines. SVs were annotated with Ensembl’s VEP [103] focusing on variants affecting protein-coding genes with the maximum SV size set to 200 Mb.

Functional classification was conducted after thorough literature and database search (OrthoDB v10 [104], Uniprot [107], NCBI Entrez gene [105]), plus Gene Ontology enrichment analysis (Shiny GO [106], false discovery rate [FDR] < 0.05). To further minimize false positives, SV calls overlapping gaps and high coverage regions (>80×) in the reference genome assembly were filtered out.

### Population genetics analysis

Genetic structure among all 150 samples was assessed using HC analysis and genetic admixture. HC was computed using SNPRelate v1.22.0 [135]. The ape v5.0 package was used for visualization of HC results [136]. Genetic admixture was estimated with ADMIXTURE v1.3.0 [137] after transforming the Variant Calling File (VCF) file into a BED file using PLINK v2.00a2LM [138, 139]. Linkage disequilibrium (LD) was evaluated after thinning the main VCF file with vcftools v0.1.13 [140] retaining sites at least 100 kb apart and then calculating *r*^2^ within windows of 5 Mb using PLINK v2.00a2LM [138, 139]. Runs of homozygosity were estimated for each sample using the RoH extension [141] in SAMtools/BCFtools v1.5 [122]. For this, allele frequencies at each SNP site and a constant recombination rate (average recombination rate mouse genome: 0.51 cM/Mb [142, 143]) were provided. These parameters, plus the genotype likelihoods stored in the VCF containing the sample, allow to identify RoHs using a hidden Markov model.

### Genetic differentiation and diversity analysis

The genomes of the trait-selected lines were compared to the neutrally evolving control line (FZTDU). For this, genetic differentiation was estimated using the *F*_ST_ index [144] in sliding window mode (size = 50 kb, step = 25 kb, min 10 SNPs) using vcftools v0.1.13 [140]. Since *F*_ST_ calculations are based on allele counts and not read counts, differences in depth between low and high coverage samples are not expected to have a direct effect in the estimation of genetic differentiation. The average number of SNP sites per window was ~ 125 (Additional file 3: Table S11). At each window, the arithmetic mean of the SNP-specific *F*_ST_ was calculated and then transformed into *z*-scores to represent its departure from the genomic mean. Additionally, all samples of the two fertility lines (DUK and DUC) were combined (pseudo-line: FERT) and compared to FZTDU as well. Since autosomes and the X-chromosomes have different effective population sizes, the X-chromosome was standardized individually. In order to identify RDDs, *F*_ST_ windows appearing simultaneously in the 95th percentile of a given contrast and in the bottom 10th percentile of all other contrast were identified. These thresholds are not derived from modelling neutrality, rather they were chosen empirically based on a previous study [51] and after testing multiple combinations of ≥95th percentiles and ≤10th percentiles, choosing the combination in which RDDs could be found in all contrasts. The upper threshold is suitable to evaluate genetic differentiation [49, 145, 146], while the bottom threshold ensures that there is practically no genetic differentiation between any of the other trait-selected lines and the control line (Fig. 6D and Fig. 7D). Genome-wide diversity patterns were assessed by measuring the nucleotide diversity (*π*) [147] in sliding windows of 50 kb size (step size = 25 kb) using vcftools v0.1.13 [140].

### Gene annotation and enrichment analysis

Genes overlapping RDDs were identified using GenomicRanges [148] and Ensembl 93’s [120] *Mus musculus* gene set. In order to sort out the most relevant genes for each of the selected traits, thorough inspection of functional annotations, literature and SNP effects was conducted. This also included testing for enrichment of Gene Ontology Biological Processes (GOBP) [149, 150] and Kyoto Encyclopedia of Genes and Genomes (KEGG) pathways [151–153] using WebGestalt [154–157] using the whole genome as reference set. A FDR threshold of 10% was used as cutoff for significant enrichment of a term or pathway. Finally, genes in quantitative trait loci (QTLs) were identified by finding overlaps with QTL data compiled in the Mouse Genome Database [158, 159].

### Data handling and visualization

Data processing and visualizations were done using R [160] and the tidyverse package [161].

## Supplementary Information

**Additional file 1.** Establishment of the Dummerstorf mouse lines, Structural Variant Calling.

**Additional file 2: Figure S1.** Response to selection throughout the selection experiment. **Figure S2.** Proportion of litters supplying parents for the next generation. **Figure S3.** Distribution of INDEL lengths. **Figure S4.** Number of private and shared INDELs among lines. **Figure S5.** SNP allele frequency state classification. **Figure S6.** Alternative allele frequency distribution. **Figure S7.** Nucleotide diversity (π) distribution in the Dummerstorf mouse lines. **Figure S8.** Example of one chromosome representative of the level of genetic diversity observed in the Dummerstorf mouse lines. **Figure S9.** Allele frequency heatmap of non-synonymous mutations in RDD genes. **Figure S10.** Allele frequency heatmap of non-synonymous mutations in RDD genes.

**Additional file 3: Table S1.** Alternative names of the Dumemrstorf mouse lines. **Table S2.** Number of SNP and INDEL sites discovered in each line. **Table S3.** Number of private variants with predicted high/moderate effects according to SnpEff. **Table S4.** Counts per length up to the 90% most frequent INDELs sorted in decreasing order of frequency. **Table S5.** Significantly enriched terms based on RDD gene lists. **Table S6.** Proportion of line-specific fixed and polymorphic structural variants in genic regions. **Table S7.** Types and lengths of line-specific fixed structural variants in genic regions. **Table S8.** Number of genes affected by line-specific fixed and polymorphic structural variants. **Table S9.** Number of genes in functional groups affected by line-specific structural variants. **Table S10.** Summary of structural variants detected in low and high coverage variant calling sets for each mouse line. **Table S11.** Number of SNP sites per window analysed with FST.

**Additional file 4: Data S1.** WGS data overview**. Data S2.** Significantly enriched terms for genes affected by line-specific SNPs and/or INDELs with high/moderate impact. **Data S3.** Genomic information of regions of distinct genetic differentiation for DUK. **Data S4.** Genomic information of regions of distinct genetic differentiation for DUC. **Data S5.** Genomic information of regions of distinct genetic differentiation for DU6. **Data S6.** Genomic information of regions of distinct genetic differentiation for DU6P. **Data S7.** Genomic information of regions of distinct genetic differentiation for DUhLB. **Data S8.** Genomic information of regions of distinct genetic differentiation for FERT. **Data S9.** Genes in regions of line-specific genetic differentiation associated to increased fertility (DUK). **Data S10.** Genes in regions of line-specific genetic differentiation associated to increased fertility (DUC). **Data S11.** Genes in regions of line-specific genetic differentiation associated to increased body mass (DU6). **Data S12.** Genes in regions of line-specific genetic differentiation associated to increased lean body mass (DU6P). **Data S13.** Genes in regions of line-specific genetic differentiation associated to increased treadmill endurance (DUhLB). **Data S14.** Genes in regions of specific genetic differentiation for the pseudo-line FERT (DUK and DUC combined). **Data S15.** Gene-spanning line-specific structural variants.

## Data Availability

The datasets generated and analysed during the current study are available in the European Nucleotide Archive (raw sequencing data; accession: PRJEB44248 [162]) and in the European Variation Archive (variant calling files; accession: PRJEB45961 [163]). Scripts used to generate the results of this publication are available in [164].

## Abbreviations

FZT: Forschungszentrum für Tierproduktion Dummerstorf
FBN: Research Institute for Farm Animal Biology
DUK: Fertility mouse line 1
DUC: Fertility mouse line 2
DU6: Body mass mouse line
DU6P: Protein mass mouse line
DUhLB: Treadmill performance mouse line
FZTDU: Control mouse line
BP: Breeding pairs
WGS: Whole Genome Sequencing
SNP: Single-nucleotide polymorphism
INDEL: Insertion deletion
RoH: Runs of homozygosity
LD: Linkage disequilibrium
HC: Hierarchical clustering
*F*_ST_: Genetic differentiation index
RDD: Region of distinct genetic differentiation
SV: Structural variant
DEL: Deletion
DUP: Duplication
INV: Inversion
SIFT: Sorting Intolerant From Tolerant
FERT: Fertility pseudo-population composed of DUK and DUC
BLUP: Best linear unbiased prediction
VQSR: Variant Quality Score Recalibration
MGP: Mouse Genomes Project
GQ: Genotype quality
DP: Genotype depth
BAM: Binary Alignment Map
VCF: Variant Calling File
VEP: Ensembl Variant Effect Predictor
MAPQ: Mapping quality
FDR: False discovery rate
GOBP: Gene Ontology Biological Processes
KEGG: Kyoto Encyclopedia of Genes and Genomes
QTL: Quantitative trait loci
