# Supplementary material for: Genomic characterization of the world’s longest selection experiment in mouse reveals the complexity of polygenic traits
Source: BMC Biol. 2022 Feb 21;20:52. doi: 10.1186/s12915-022-01248-9 (PMC8862358; doi:10.1186/s12915-022-01248-9)
Supplement: Supplementary file 1 — Additional file 1. Establishment of the Dummerstorf mouse lines, Structural Variant Calling. [file 12915_2022_1248_MOESM1_ESM.docx]

**Supplementary Methods**

1. **Establishment of the Dummerstorf mouse lines**

During the years 1969 and 1970, four outbred strains (NMRI orig., Han:NMRI, CFW, CF1) and four inbred strains (CBA/Bln, AB/Bln, C57BL/Bln, XVII/Bln) were systematically crossed to establish the line **FZTDU** (*Forschungszentrum für Tierproduktion Dummerstorf*) [5,6]. Full-sib mating was avoided by random mating. This line has been kept unselected for almost 200 generations. The line was originally maintained with 200 breeding pairs per generation until animals were moved from a conventional semi-barrier housing into a specific pathogen-free (SPF) environment on generation ~160. This transition could only be accomplished through a limited number of embryo transfers and as a result, the number of breeding pairs dropped to 55. Thereafter, the number of breeding pairs has been kept at 125 (the current number of breeding pairs) avoiding consanguinity by random mating. All the Dummerstorf selection lines were derived from FZTDU starting at different time-points.

In general, all trait-selected lines were developed through among-family selection [108], whereby litters were ranked according to each trait of interest (Table 2) and then parents were chosen at random from the highest ranked litters. The proportion of litters selected varied generation to generation (Additional file 2: Figure S2). The trait-selected lines were maintained with 60-100 breeding pairs. However, when animals had to be relocated to the new SPF animal housing building in 2011 after 120-165 generations, the number of breeding pairs drastically dropped to ~20 for DUK, DUC, DU6P and DUhLB, and as low as 7 for DU6 (Table 1).

The process of selection to establish the **fertility lines DUK and DUC** began shortly after the establishment of FZTDU. In 1971, each fertility line was started with 60 breeding pairs. These animals were drawn from FZTDU by phenotypic maternal selection for number of offspring and litter weight at birth in the first litter (for more details see [22]). However, for an interim period of 10 generations families were ranked only by litter weight. Selection for fertility continues to this day spanning more than 190 generations. The lowest number of breeding pairs was 19 (DUK) and 24 (DUC) at generation ~164 when animals were transferred to a new facility by embryo transfer. Nowadays, the number of breeding pairs is 60 for both lines. Before relocation, the number of breeding pairs per generation and the selection intensity ranged between 60% and 100% and between 25% and 45%, respectively. A few generations after relocation, family information and individual information was combined in a pedigree-based BLUP (Best Linear Unbiased Prediction) [109] estimation of breeding values and selection was conducted accordingly.

The **body mass line DU6** was started in 1975 by phenotypic selection of FZTDU by ranking litters according to total weight of two randomly sampled males from each litter at 42 days of age. Whenever possible these males were not chosen as sires. DU6 was founded by mating 80 pairs at around 9 weeks of age. The number of breeding pairs until generation ~154 was kept at 60-100 pairs. On generation ~154, animals were transferred to the new facility and the number of breeding pairs decreased to only 7 because of embryo transfer yield. Thereafter the number of breeding pairs was increased to 60 and phenotyping was massively extended by taking body weights at day 42 from all progeny, including females. The selection strategy was later changed on generation 161 to selection based on estimated breeding values. The breeding values for body mass at day 42 were calculated using with BLUP [109]. As of generation 173 the proportion of female to male breeders has been kept at 2:1 (120 females and 60 males approx.) to mitigate the decreased pregnancy rate observed in DU6 females. This line continues to be selected (selection intensity of 45-90%).

Also in 1975, the **DU6P** line was established by selection for weight and protein content of the carcass of a single male from each litter at 42 days of age Occasionally protein mass could not be determined (e.g. because of technical issues or limited lab capacities), in which case litters were ranked by the combined weight of two males, as described for line DU6. The number of breeding pairs at the start of this line was 80 and was kept at 60-80 breeding pairs per generation. Then, on generation ~154, animals were relocated with 19 breeding pairs as founders, which were then increased to 60 pairs. Selection in DU6P stopped at generation 152 and the line is currently preserved without selection pressure by allowing random mating.

Finally, the high **endurance line DUhLB** was started in 1982 based on selection for high treadmill performance. It is thus the youngest of the Dummerstorf selection lines, as well as the shortest selected one (selection stopped at generation 141). Male running performance was evaluated based on distance (meters) covered on a treadmill before exhaustion (submaximal test). Trials were conducted after mating at 11 weeks of age. Subjects had no previous access to any kind of equipment that would influence their performance (untrained). Offspring of the highest scoring subjects were chosen for breeding. The line was founded with 100 breeding pairs and a selection intensity of 40% for the first 25 generations. Thereafter, the line was maintained with 60-80 breeding pairs at a selection intensity of 45-100%. On generation ~120, 44 breeding pairs were used as founders after transferring to the new facility. Together with DU6P, DUhLB is currently preserved without selection.

1. **Structural Variant Calling**

Mapped and deduplicated short PE reads were used in detection of structural variants. As depth of coverage of reads mapped to the reference mouse genome sequence varied between 5 and 20x, we have split samples for each line into a high (10 samples) and low coverage (15 samples) set, and conducted structural variation analysis separately on these sets.

Three SV callers, Manta v.1.6.0 [110], Whamg v.1.7.0 [111] and Lumpy v.0.2.13 [112] were selected based on their sensitivity and precision [113]. Manta integrates paired-read (PR), split-read (SR) evidence and SV breakend assembly (AS) during SV discovery. Whamg implements PR and SR support while Lumpy relies on a probabilistic copy number variation discovery combing PR, SR and read-depth (RD). Intersecting results of multiple SV callers applying different SV detection approaches has previously been shown to improve accuracy of variant call sets [113,114].

Manta SV calls in genomic regions with depth greater than 3x the median chromosome depth near one or both SV breakends mainly caused by reads mapping in low complexity regions, as well as reads with MAPQ<30 were filtered out. Furthermore, variant calls for which samples did not pass Manta caller quality filters, and have genotype quality below 20 (GQ<20) have been filtered out. Calls with paired-read (PR) and split-read (SR) support of PR>=3 and SR>=3 were retained.

Whamg SV calls of size <50bp and >2Mb were filtered out, along with calls with fewer than 5 supporting reads and GQ<20. Calls associated with poorly mapped regions and BND-type calls with high cross-chromosomal mapping scores (CW>0.2) were removed, as Whamg does not specifically call translocations. Lumpy calls for which evidence supporting the variant were below 5 (SU<5) and calls with GQ<20 were filtered out. Both Whamg and Lumpy SV call sets were genotyped with Svtyper v0.7.1 [101].

Unlocalized and unplaced scaffolds have been removed from all SV sets and only scaffolds assigned to chromosomes have been included in further analysis. Survivor v.1.0.7 [102] was used to merge SV call sets within and among samples. For each mice line sample, we first merged SV events of the same type, called by at least two SV callers, with start/ end positions detected within +/-1000 bp, identified in at least 60% of samples in a low coverage set (10 samples out of 15) and 100% of samples in a high coverage set (all 10 samples) for each mice line.

The union of SVs detected in two separate sample sets for each line were further used. We then intersected SV calls among all mice lines to obtain SVs private for each mice line (line-specific) and shared among lines (Additional file 2: Figure S11). To further reduce the FDR, SV calls overlapping gaps and high coverage regions (> 80x) in the reference genome assembly were filtered out. High coverage regions were determined for each mouse line based on the intersection among samples of 1-kb windows containing reads mapping with depth of coverage > 80x. Variants specified as “BND” (translocations) were removed and deletions, and duplications and inversions were further investigated (Additional file 2: Figure S12-S15).

We annotated the final SV set with the Ensembl VEP v. 101.0 [103] focusing on variants overlapping protein-coding genes (maximum SV size = 200 Mb). Functional classification of genes was based on literature and database search (OrthoDB v10 [104]; Uniprot [107]; NCBI Entrez gene [105]), and Gene Ontology enrichment analysis (Shiny GO, FDR < 0.05 [106]).
