# Supplementary material for: Genomic characterization of the world’s longest selection experiment in mouse reveals the complexity of polygenic traits
Source: BMC Biol. 2022 Feb 21;20:52. doi: 10.1186/s12915-022-01248-9 (PMC8862358; doi:10.1186/s12915-022-01248-9)
Supplement: Supplementary file 3 — Additional file 3: Table S1. Alternative names of the Dumemrstorf mouse lines. Table S2. Number of SNP and INDEL sites discovered in each line. Table S3. Number of private variants with predicted high/moderate effects according to SnpEff. Table S4. Counts per length up to the 90% most frequent INDELs sorted in decreasing order of frequency. Table S5. Significantly enriched terms based on RDD gene lists. Table S6. Proportion of line-specific fixed and polymorphic structural variants in genic regions. Table S7. Types and lengths of line-specific fixed structural variants in genic regions. Table S8. Number of genes affected by line-specific fixed and polymorphic structural variants. Table S9. Number of genes in functional groups affected by line-specific structural variants. Table S10. Summary of structural variants detected in low and high coverage variant calling sets for each mouse line. Table S11. Number of SNP sites per window analysed with FST. [file 12915_2022_1248_MOESM3_ESM.docx]

**Supplementary tables**

| **Table S1. Alternative names of Dummerstorf mouse lines and reference list of scientific articles based on these mouse lines. Status: November 2021** | |
| --- | --- |
| **Line** | **Alternative Name and Reference** |
| DUK | DU-K [37], FL1 [20,22-24,38-41] |
| DUC | DU-C [37], FL2 [20,22,38,40] |
| DU6 | BW [25,26], Titan [21] |
| DU6P | PA [25] |
| DUhLB | DU-hTP [10,27-32] |
| FZTDU | Fzt: DU [6,8,25,26,32,37], DUK [34-36], Ctrl [22,23] |

| **Table S2. Number of SNP and INDEL sites discovered in each line** | | | | |
| --- | --- | --- | --- | --- |
| **Line** | **SNPs** | **% in FZTDU** | **INDELs (Insertions + Deletions)** | **% in FZTDU** |
| DUK | 2,305,349 | 92.76 | 338,380 (166,838 + 171,542) | 90.41 |
| DUC | 2,615,584 | 92.76 | 376,453 (185,791 + 190,662) | 91.20 |
| DU6 | 2,744,788 | 93.18 | 396,022 (195,415 + 200,607) | 90.97 |
| DU6P | 2,899,902 | 91.04 | 417,027 (206,164 + 210,863) | 89.52 |
| DUhLB | 3,196,655 | 92.26 | 455,789 (225,415 + 230,374) | 90.65 |
| FZTDU | 4,453,865 | -- | 638,500 (315,851 + 322,649) | -- |

| **Table S3. Number of private variants with predicted high/moderate effects according to SnpEff.** | | | |
| --- | --- | --- | --- |
|  | **SNPs** | **INDELs** | **Genes** |
| DUK | 996 | 92 | 517 |
| DUC | 640 | 101 | 465 |
| DU6 | 752 | 127 | 546 |
| DU6P | 783 | 109 | 534 |
| DUhLB | 1970 | 176 | 1027 |
| Private variants for FZTDU not included as this line was unselected. | | | |

| **Table S4. Counts per length up to the 90% most frequent INDELs sorted in decreasing order of frequency** | | | | |
| --- | --- | --- | --- | --- |
|  | | | | |
| **LENGTH** | **COUNT** | **PRCT** | **CLASS** | **CUMSUM_PRCT** |
| -1 | 174467 | 22.76 | deletion | 22.76 |
| 1 | 173290 | 22.6 | insertion | 45.36 |
| -2 | 62514 | 8.15 | deletion | 53.51 |
| 2 | 59929 | 7.82 | insertion | 61.33 |
| -3 | 32609 | 4.25 | deletion | 65.58 |
| 3 | 30779 | 4.01 | insertion | 69.6 |
| -4 | 29781 | 3.88 | deletion | 73.48 |
| 4 | 29271 | 3.82 | insertion | 77.3 |
| -5 | 12315 | 1.61 | deletion | 78.91 |
| 5 | 12167 | 1.59 | insertion | 80.5 |
| 6 | 10170 | 1.33 | insertion | 81.82 |
| -6 | 9875 | 1.29 | deletion | 83.11 |
| -7 | 8919 | 1.16 | deletion | 84.27 |
| -8 | 8512 | 1.11 | deletion | 85.38 |
| 7 | 8360 | 1.09 | insertion | 86.47 |
| 8 | 7651 | 1 | insertion | 87.47 |
| -9 | 6098 | 0.8 | deletion | 88.27 |
| -10 | 5838 | 0.76 | deletion | 89.03 |
| 9 | 5403 | 0.7 | insertion | 89.73 |
| 10 | 5147 | 0.67 | insertion | 90.41 |
| -12 | 4400 | 0.57 | deletion | 90.98 |

| **Table S5. Significantly enriched terms based on RDD gene lists** | | | |
| --- | --- | --- | --- |
| Line | **Term/Pathway** | **Genes** | **FDR** |
| DUK | mmu04072: Phospholipase D signaling pathway | Raf1  Adcy6  Grm8  Tsc1  Ralgds | 0.0082875 |
| DUC | GO:0009755: hormone-mediated signaling pathway | Pias2  Pgr  Rxfp1  Yap1 | 0.030545 |
| DUC | GO:0048814: regulation of dendrite morphogenesis | Pias2  Trpc6  Skor2 | 0.074441 |
| DUC | GO:0030518: intracellular steroid hormone receptor signaling pathway | Pias2  Pgr  Yap1 | 0.074441 |
| DUhLB | mmu00340: Histidine metabolism | Aldh3a1  Aldh3a2 | 0.099122 |
| DUhLB | mmu00410: beta-Alanine metabolism | Aldh3a1Aldh3a2 | 0.099122 |
| Significantly enriched GO terms and pathways at FDR < 0.1 | | | |

| **Table S6. Proportion of line-specific fixed and polymorphic structural variants in genic regions** | | | | | | |
| --- | --- | --- | --- | --- | --- | --- |
|  |  | **Fixed** | |  | **Polymorphic** | |
|  |  | **Number** | **Length (mean)** |  | **Number** | **Length (mean)** |
| DUK |  | 5 | 13.58 (2.7) kbp |  | 15 | 204.62 (13.64) Mb |
| DUC |  | 2 | 2.19 kbp (1.1 kbp) |  | 34 | 186.92 (5.5) Mb |
| DU6 |  | 4 | 7.75 (1.94) kbp |  | 14 | 302 (21.57) kb |
| DU6P |  | 7 | 29.11 (4.65) kbp |  | 17 | 129.96 (7.6) Mb |
| DUhLB |  | 6 | 11.16 (1.9) kbp |  | 14 | 3.72 (0.265) Mb |
| FZTDU |  | 1 | 1.14 kbp |  | 8 | 14.09 (1.8) Mb |

| **Table S7. Types and lengths of line-specific fixed structural variants in genic regions** | | | | | | |
| --- | --- | --- | --- | --- | --- | --- |
|  | **DEL** | **DEL length** | **DUP** | **DUP length** | **INV** | **INV length** |
| DUK | 5 | 13.6 Kb | -- | -- | -- | -- |
| DUC | 1 | 1.3 Kb | -- | -- | 1 | 0.923 Kb |
| DU6 | 4 | 7.7 Kb | -- | -- | -- | -- |
| DU6P | 3 | 8.5 Kb | 1 | 11 Kb | 3 | 9.6 Kb |
| DUhLB | 4 | 3.3 Kb | -- | -- | 2 | 7.9 Kb |
| FZTDU | 1 | 1.1 Kb | -- | -- | -- | -- |

| **Table S8. Number of genes affected by line-specific fixed and polymorphic structural variants** | | | | | | | |
| --- | --- | --- | --- | --- | --- | --- | --- |
|  | **Fixed** | | |  | **Polymorphic** | | |
|  | **DEL** | **DUP** | **INV** |  | **DEL** | **DUP** | **INV** |
| DUK | 5 | -- | -- |  | 4 | 28 | 1694 |
| DUC | 1 | -- | 1 |  | 9 | 38 | 1363 |
| DU6 | 4 | -- | -- |  | 6 | -- | 7 |
| DU6P | 3 | 1 | 3 |  | 11 | -- | 1130 |
| DUhLB | 4 | -- | 2 |  | 11 | 3 | 7 |
| FZTDU | 1 | -- | -- |  | 3 | -- | 266 |

| **Table S9. Number of genes in functional groups affected by line-specific structural variants** | | | | | | |
| --- | --- | --- | --- | --- | --- | --- |
|  | **DUK** | **DUC** | **DU6** | **DU6P** | **DUhLB** | **FZTDU** |
| **Reproduction** | 8 | 13 | 1 | 1 | 3 | 1 |
| **Metabolism/Energy conversion** | -- | 27 | 3 | 16 | 8 | -- |
| **Immune system** | -- | 3 | 1 | 4 | 1 | 13 |
| **Nervous system** | 5 | 13 | 1 | 3 | 4 | 1 |
| **Cardiovascular system** | 2 | 1 | 1 | 1 | 2 | 15 |
| **Endocrine system** | 2 | 2 | 2 | 1 | 2 | -- |
| **Sensory perception** | 297 | 36 | 2 | -- | 3 | -- |
| **Other (cell cycle, transcription)** | 2 | 88 | 3 | 15 | 1 | 2 |

| **Table S10. Summary of structural variants detected in low and high coverage variant calling sets for each mice line.** | | | | | | | | | |
| --- | --- | --- | --- | --- | --- | --- | --- | --- | --- |
|  | **Low-coverage set** | | | |  | **High-coverage set** | | | |
|  | **DEL** | **DUP** | **INV** | **Total** |  | **DEL** | **DUP** | **INV** | **Total** |
| DUK | 1965 | 11 | 81 | 2057 |  | 3548 | 31 | 515 | 4094 |
| DUC | 1693 | 6 | 36 | 1735 |  | 4897 | 51 | 1241 | 6189 |
| DU6 | 63 | 3 | 5 | 71 |  | 5014 | 28 | 554 | 5596 |
| DU6P | 2735 | 10 | 104 | 2849 |  | 2716 | 21 | 2063 | 4800 |
| DUhLB | 1992 | 6 | 87 | 2085 |  | 3755 | 21 | 1481 | 5257 |
| FZTDU | 2408 | 6 | 107 | 2521 |  | 2454 | 12 | 540 | 3006 |

| **Table S11. Number of SNP sites per window analyzed with F_ST_** | | | | | |
| --- | --- | --- | --- | --- | --- |
| **Contrast** | **Number of**  **windows** | **Mean**  **sites/window** | **SD**  **sites/window** | **Min sites/window** | **Max**  **sites/window** |
| DUK_ FZTDU | 70621 | 125.03 | 115.02 | 10 | 1274 |
| DUC_ FZTDU | 71172 | 124.42 | 114.54 | 10 | 1274 |
| DU6_ FZTDU | 71036 | 124.16 | 115.1 | 10 | 1274 |
| DU6P_ FZTDU | 71633 | 125.71 | 115.51 | 10 | 1274 |
| DUhLB_ FZTDU | 71222 | 125.38 | 115.45 | 10 | 1274 |
| FZTDU_ FZTDU | 72702 | 125.99 | 115.88 | 10 | 1274 |
