# Supplementary material for: Author Correction: Genomic characterization of the world’s longest selection experiment in mouse reveals the complexity of polygenic traits
Source: BMC Biol. 2022 Oct 20;20:238. doi: 10.1186/s12915-022-01439-4 (PMC9585779; doi:10.1186/s12915-022-01439-4)
Supplement: Supplementary file 1 — Additional file 2: Figure S1. Response to selection throughout the selection experiment. Data points indicate the trait-value at each generation for the trait-selected lines and their controls. The first measure taken after relocation to a new mouse house is indicated by a red diamond shape surrounding the data point. Trend lines and confidence intervals are based on local regression (locally estimated scatterplot smoothing). For the case of the protein-mass line DU6P, measurements were not collected after relocation. Though FZTDU can be considered the control line, as it has been evolving neutrally over the span of the breeding experiment, other lines have been used as controls in the past and are indicated in the figure as “Duks” (specific control line for the body (DU6) and protein (DU6P) mass lines) and “DUKB” (specific control line for the treadmill performance line DUhLB). None of these line-specific control lines exist anymore and for the case of “Duks”, data is incomplete. Figure S2. Proportion of litters supplying parents for the next generation. Proportions varied according to the selection intensities required by the breeding program over the last ~50 years. Indicated by a diamond shape are the data points corresponding to the first generation after relocation to a new mouse house. For the case of the protein-mass line DU6P, there was no data after relocation. Figure S3. Distribution of INDEL lengths. (A) INDEL length distribution comprising ~90% of INDELs. (B) Total number of insertions and mutations (392,051 deletions and 374,604 insertions). Only INDELs outside microsatellites were considered. Figure S4. Classification of INDELs by type, fixation and presence in control line. INDEL sites were classified as fixed or not-fixed if their allele frequencies were 1 or <1, respectively. At each line, the fraction of INDELs shared (in FZTDU, blue) and not shared (not in FZTDU, red) with FZTDU is also shown. INDELs overlapping microsatellite regions were remove [file 12915_2022_1439_MOESM1_ESM.docx]

**Supplementary figures**

**
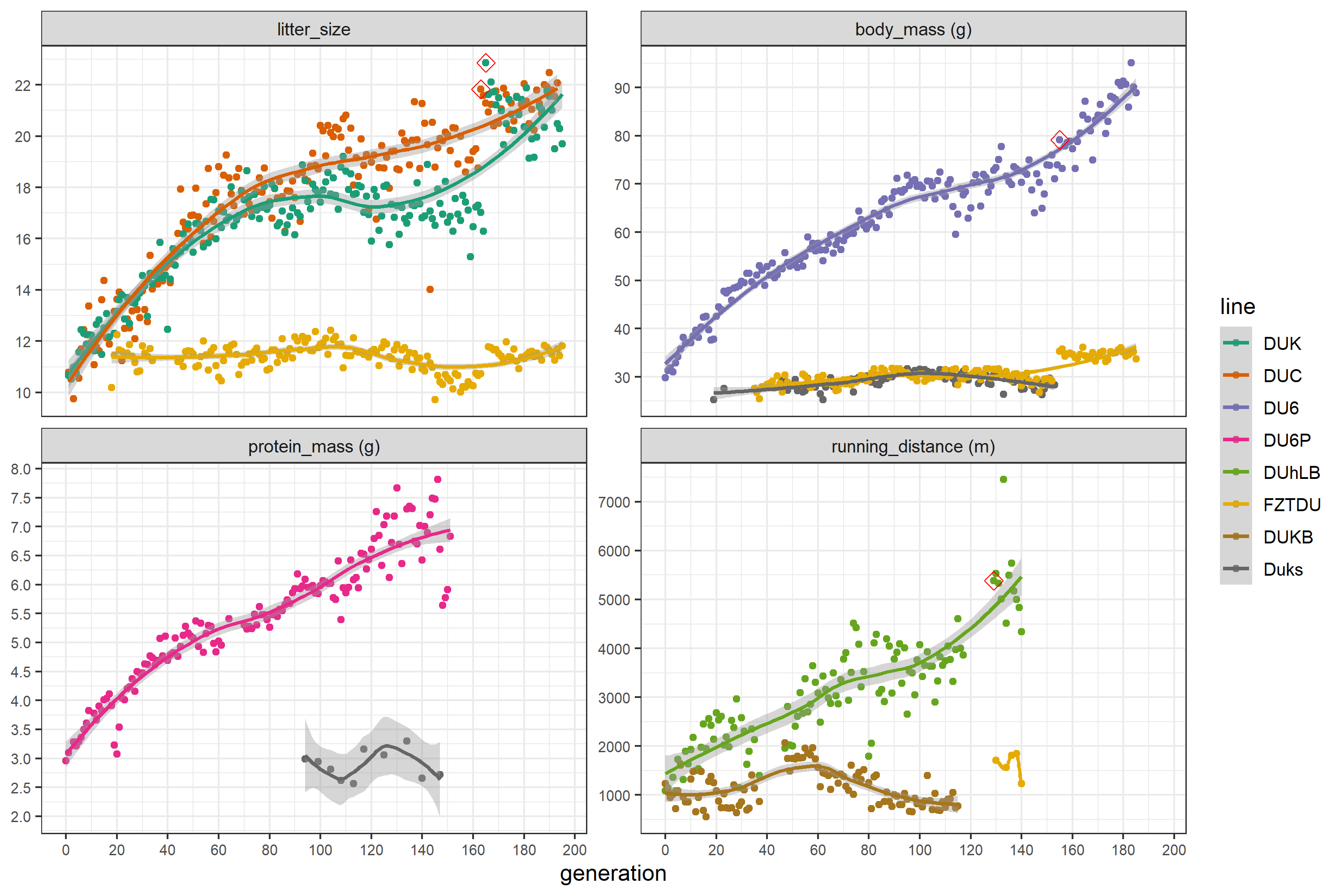
**

**Figure S1. Response to selection throughout the selection experiment.** Data points indicate the trait-value at each generation for the trait-selected lines and their controls. The first measure taken after relocation to a new mouse house is indicated by a red diamond shape surrounding the data point. Trend lines and confidence intervals are based on local regression (locally estimated scatterplot smoothing). For the case of the protein-mass line DU6P, measurements were not collected after relocation. Though FZTDU can be considered the control line, as it has been evolving neutrally over the span of the breeding experiment, other lines have been used as controls in the past and are indicated in the figure as “Duks” (specific control line for the body (DU6) and protein (DU6P) mass lines) and “DUKB” (specific control line for the treadmill performance line DUhLB). None of these line-specific control lines exist anymore and for the case of “Duks”, data is incomplete.

**
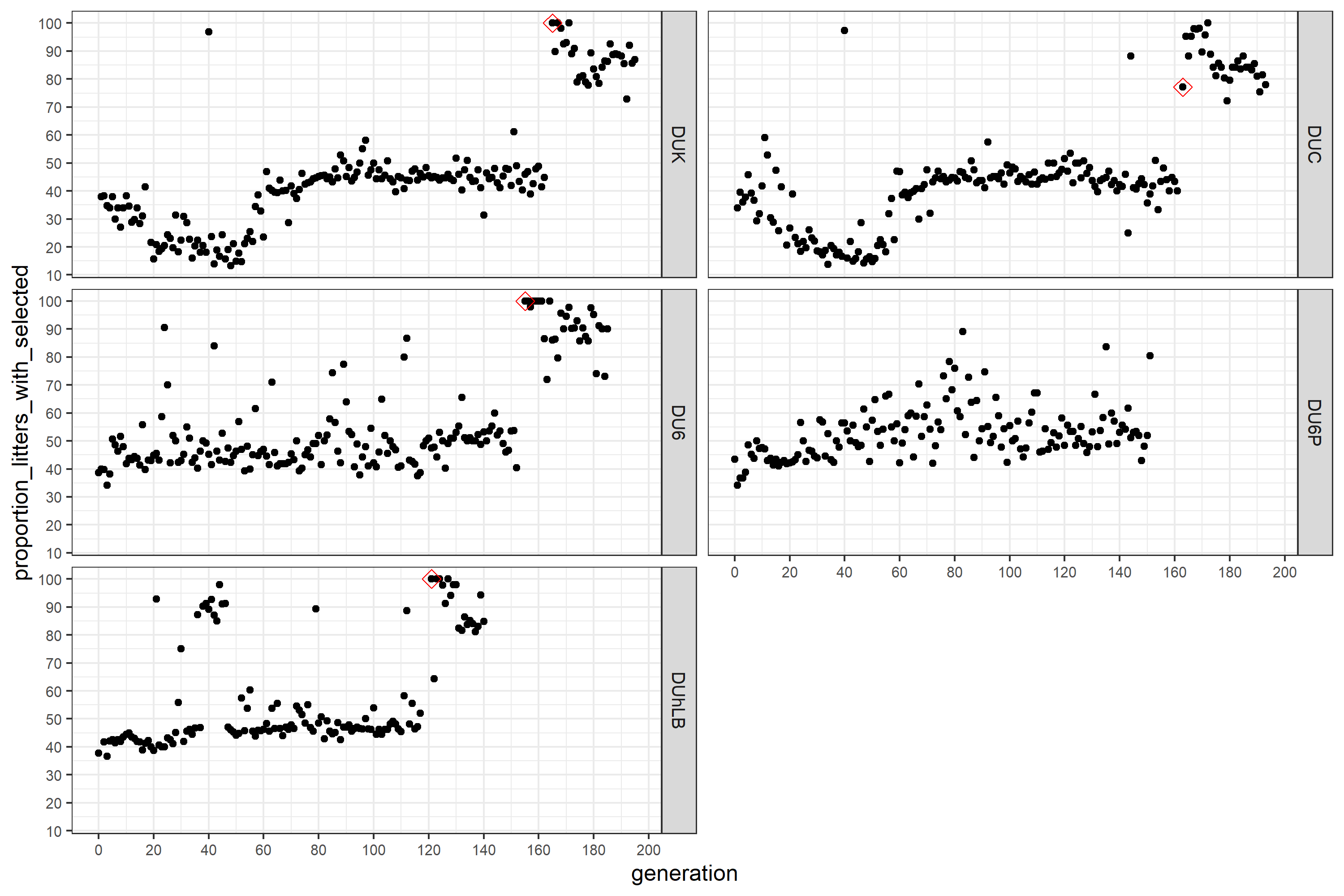
**

**Figure S2. Proportion of litters supplying parents for the next generation.** Proportions varied according to the selection intensities required by the breeding program over the last ~50 years. Indicated by a diamond shape are the data points corresponding to the first generation after relocation to a new mouse house. For the case of the protein-mass line DU6P, there was no data after relocation.

**
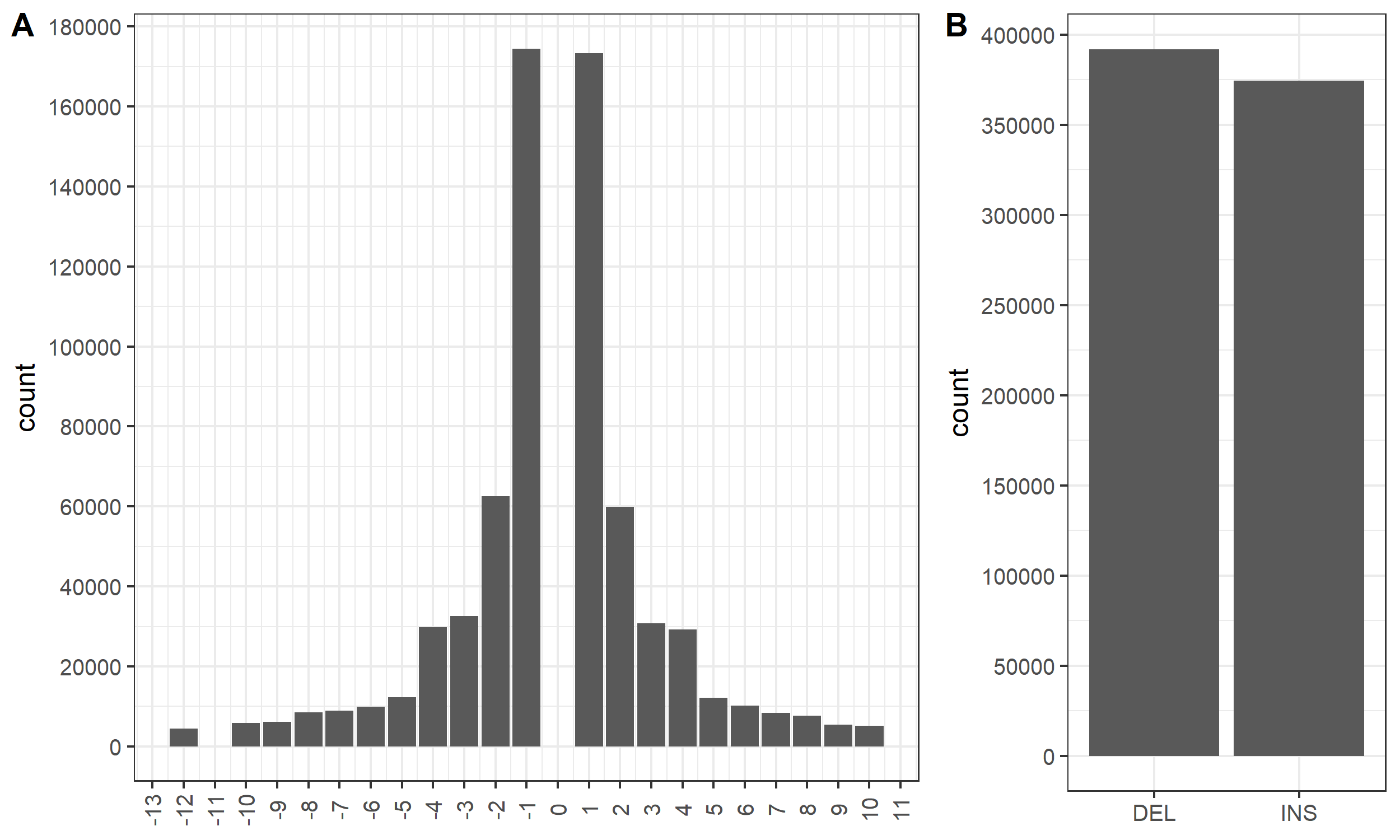
**

**Figure S3. Distribution of INDEL lengths. (A)** INDEL length distribution comprising ~90% of INDELs. **(B)** Total number of insertions and mutations (392,051 deletions and 374,604 insertions). Only INDELs outside microsatellites were considered.

**
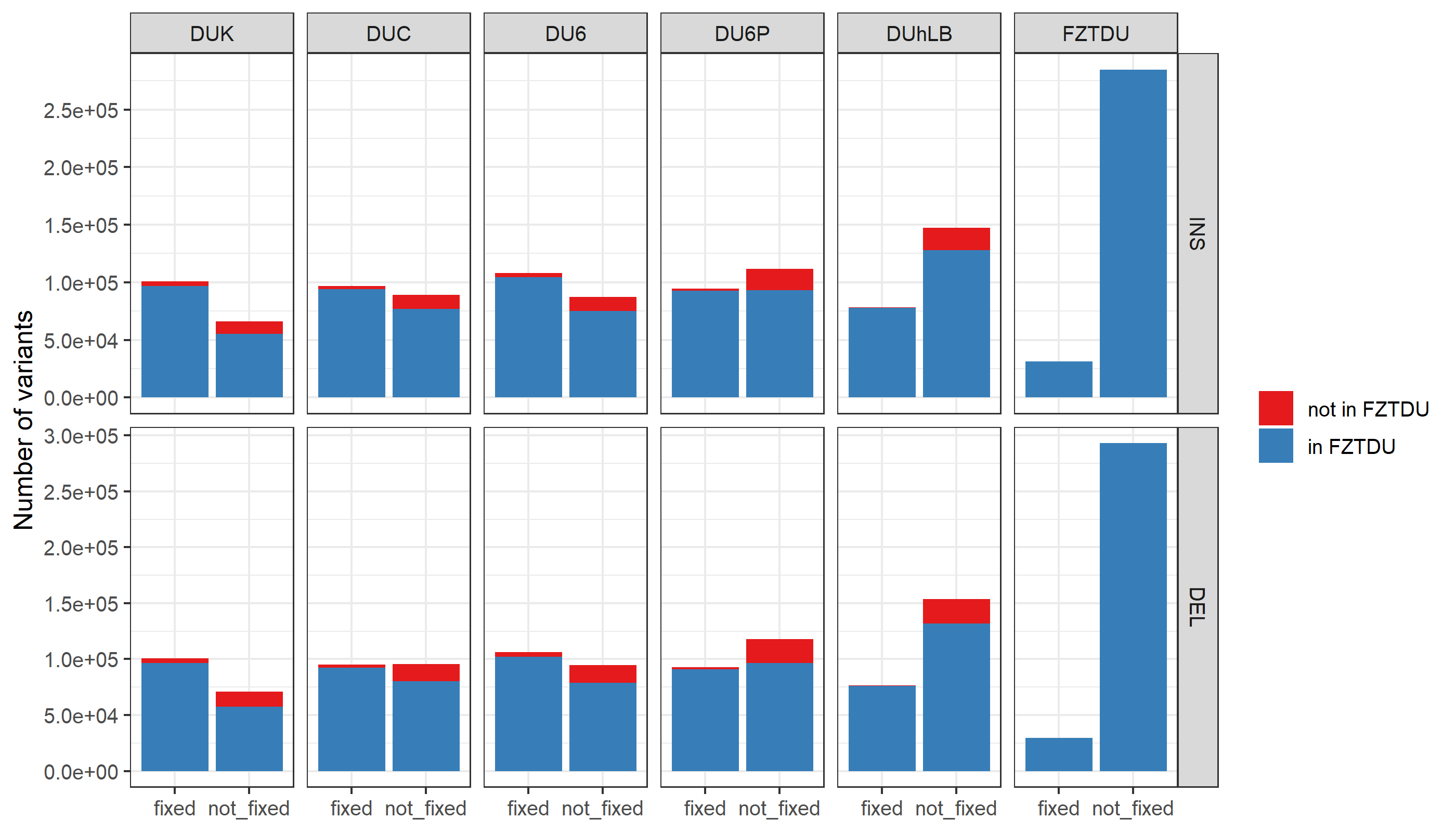
**

**Figure S4. Classification of INDELs by type, fixation and presence in control line.** INDEL sites were classified as fixed or not-fixed if their allele frequencies were 1 or <1, respectively. At each line, the fraction of INDELs shared (in FZTDU, blue) and not shared (not in FZTDU, red) with FZTDU is also shown. INDELs overlapping microsatellite regions were removed from the analysis.

**
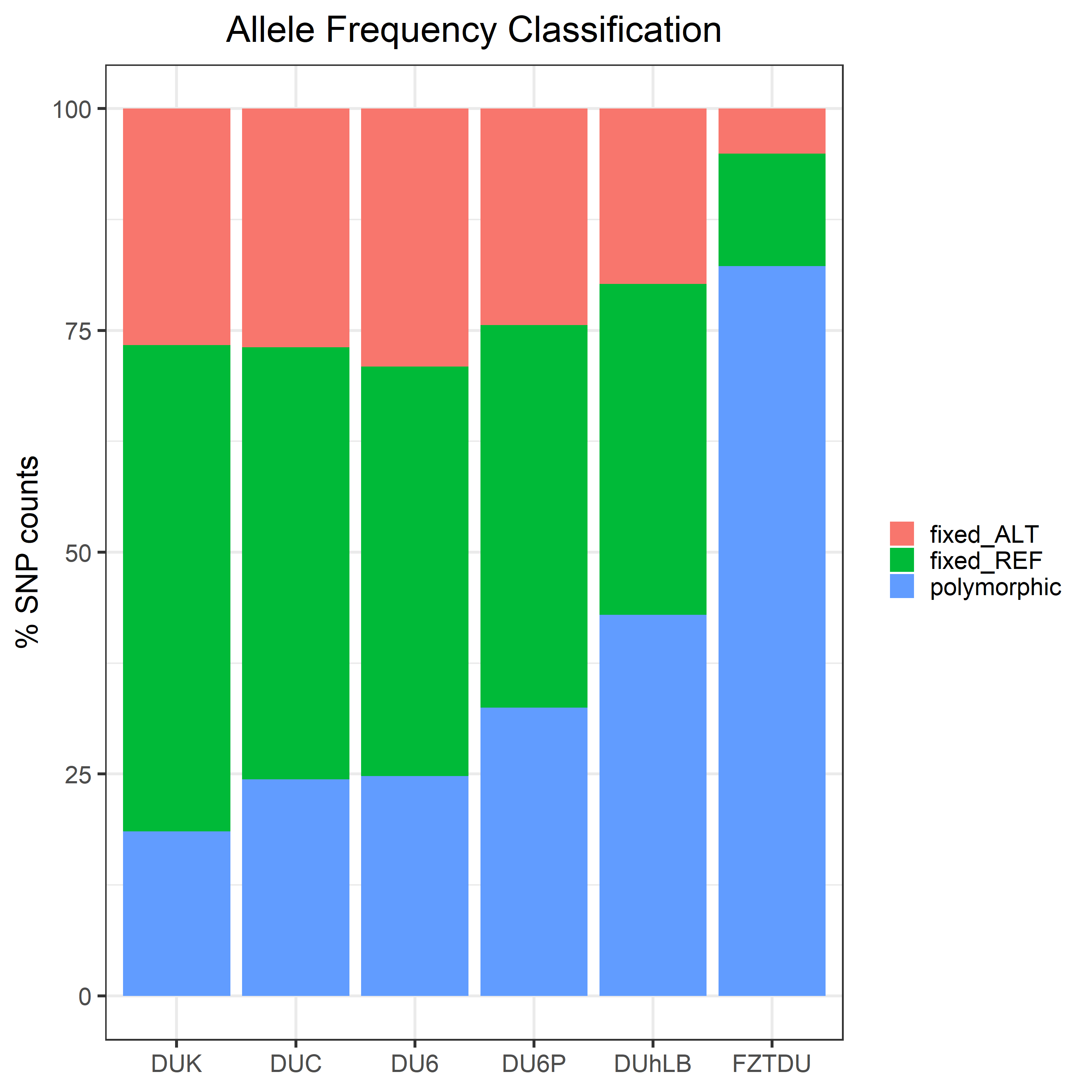
**

**Figure S5. SNP allele frequency state classification**. Sites in which SNPs were observed across the whole set of samples are classified for each mouse line as fixed-alternative (alternative allele homozygous in all subjects, fixed_ALT), fixed-reference (reference allele homozygous in all subjects, fixed_REF) and polymorphic (not fixed).


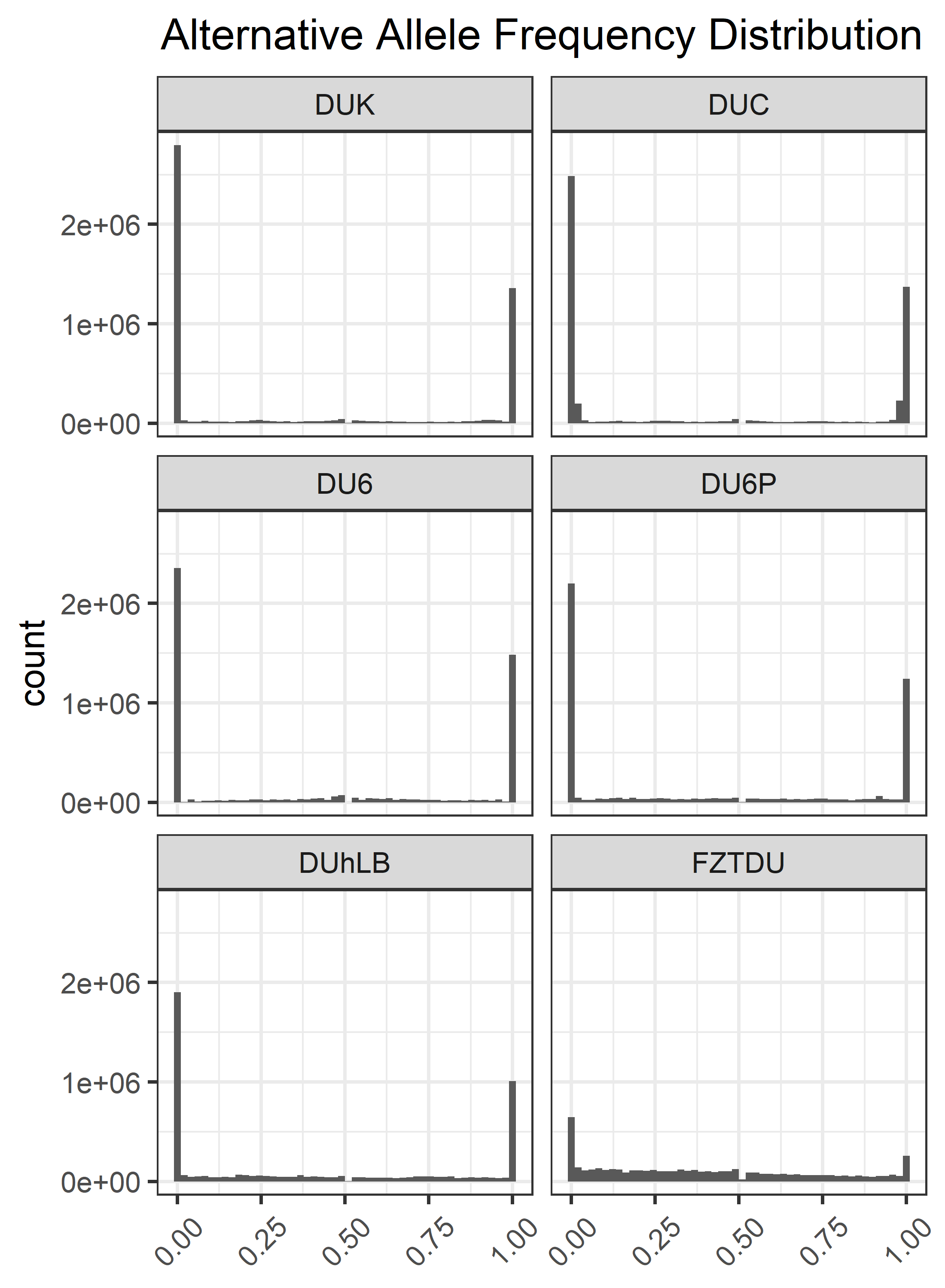


**Figure S6. Alternative allele frequency distribution.** Counts of SNPs along the allele frequency spectrum for each mouse line.


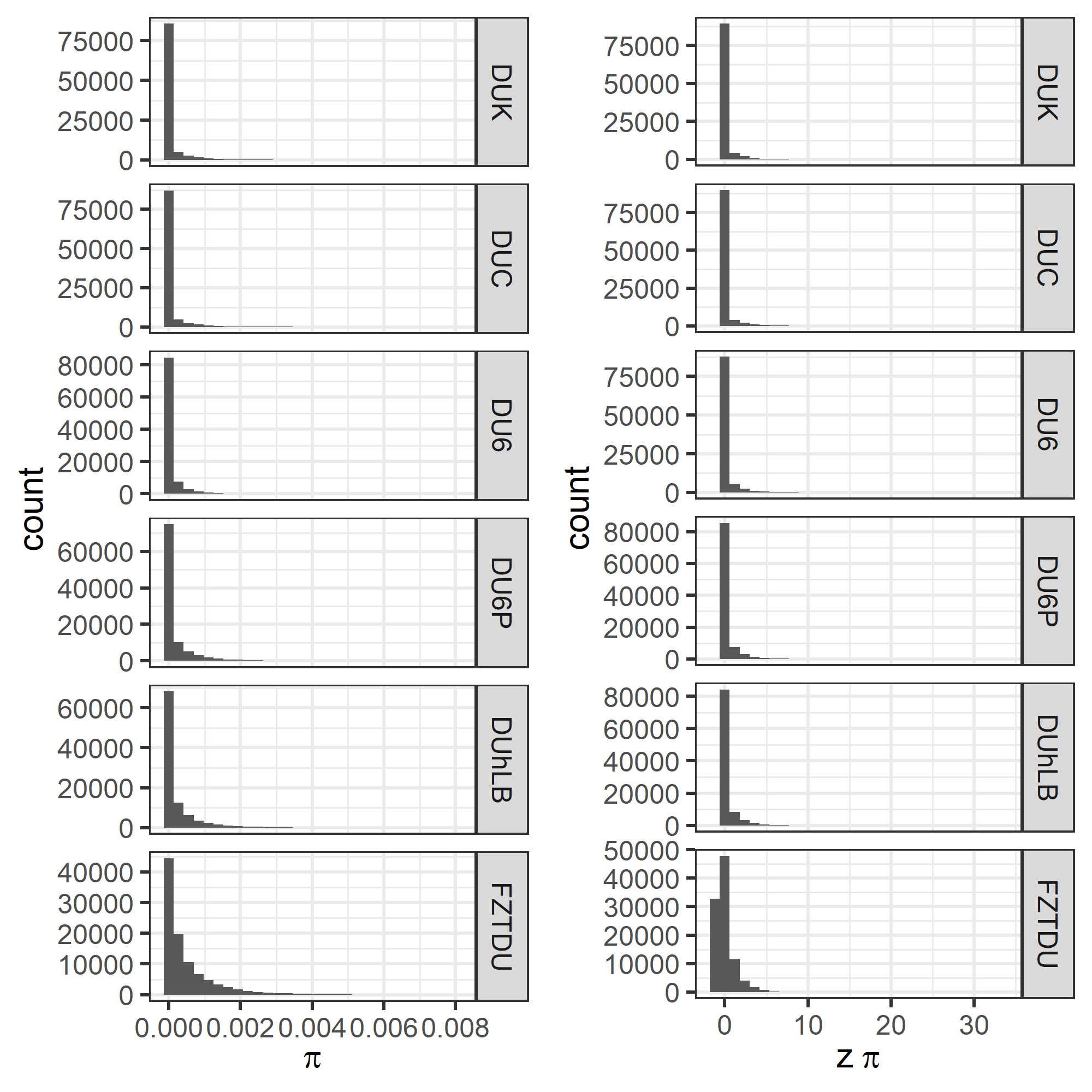


**Figure S7. Nucleotide diversity (π) distribution in the Dummerstorf mouse lines.** Nucleotide diversity (π) was calculated in sliding window mode (size=50Kb, step=25Kb, ≥10 SNPs). Scores were transformed to z-scores in order to represent the data in terms of standard deviations from the genomic mean. The distributions illustrate the low levels of genetic diversity within lines, with the most scores accumulate at the lower end of the distribution (left), thus highly diverse regions are rare events.

**
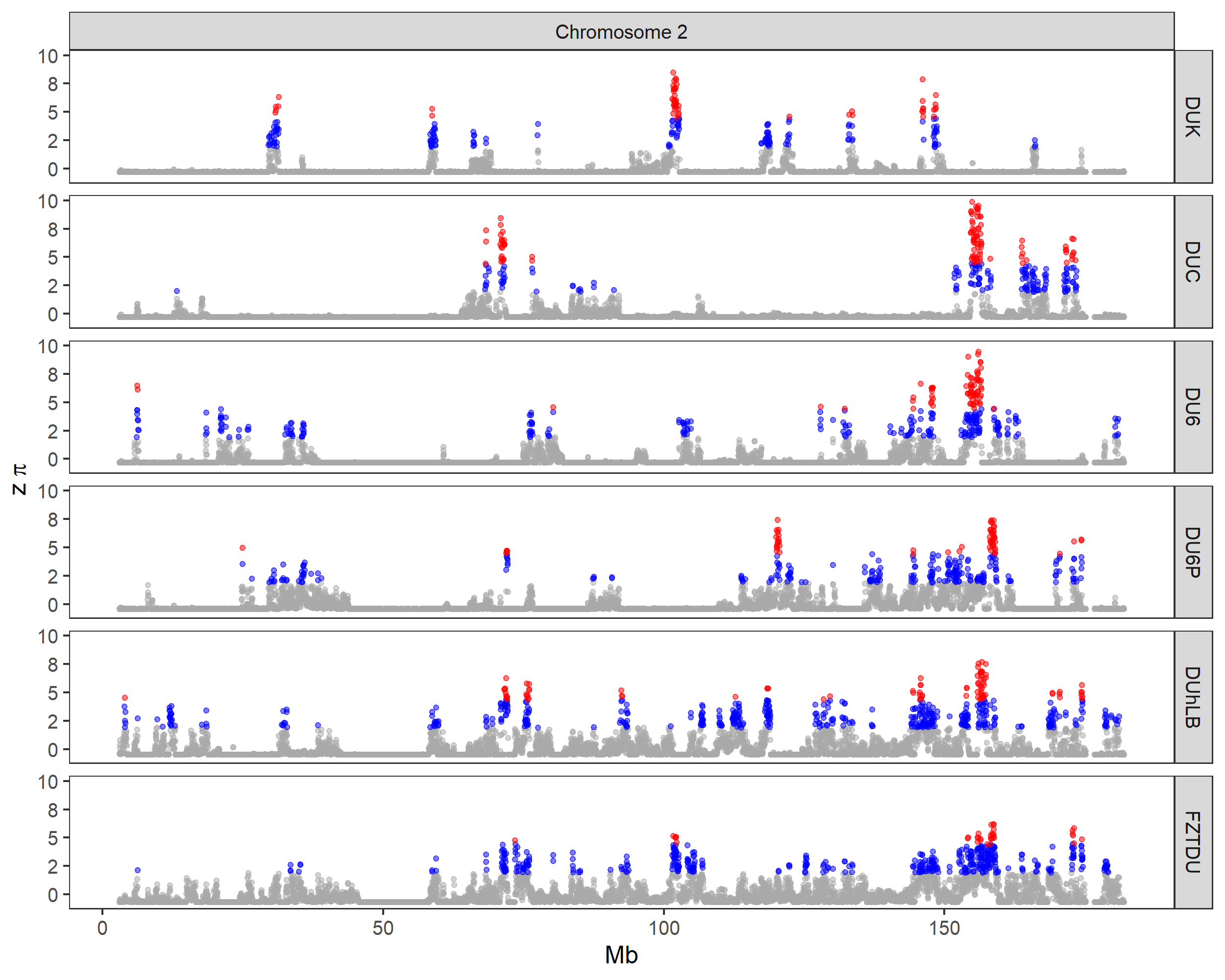
**

**Figure S8. Example of one chromosome representative of the level of genetic diversity observed in the Dummerstorf mouse lines.** The stretches of low diversity are longer and more abundant than in FZTDU. Regions of extreme genetic diversity are shown in blue (top 5% most diverse windows) and red (top 1% most diverse windows).

**
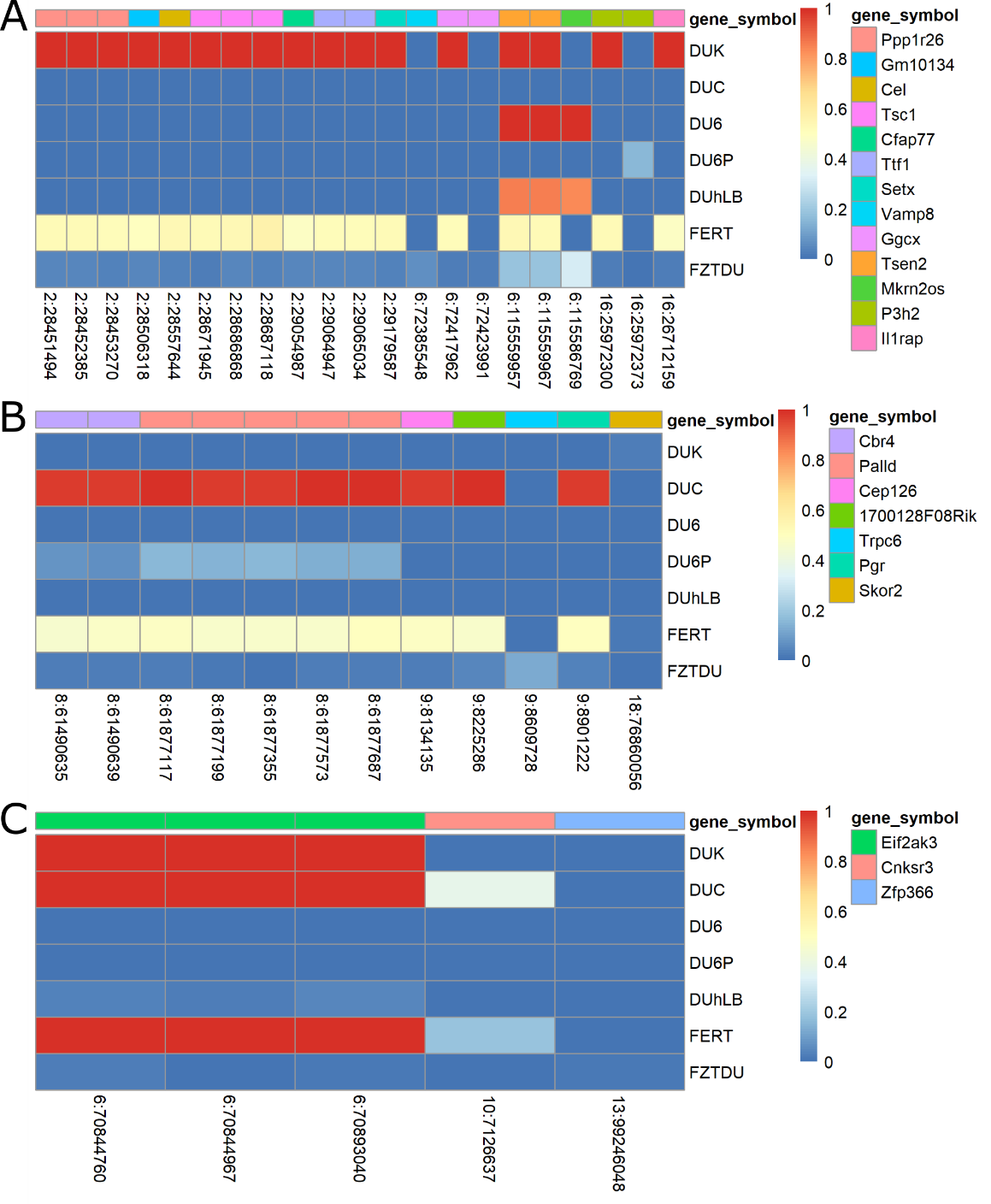
**

**Figure S9. Allele frequency heatmap of non-synonymous mutations in RDD genes.** Allele frequencies of non-synonymous SNPs in genes overlapping regions of distinct genetic differentiation for DUK (**A**), DUC (**B**) and the joint fertility population FERT (**C**). The gradient scale represents the allele frequency from low (blue) to high (red).

**
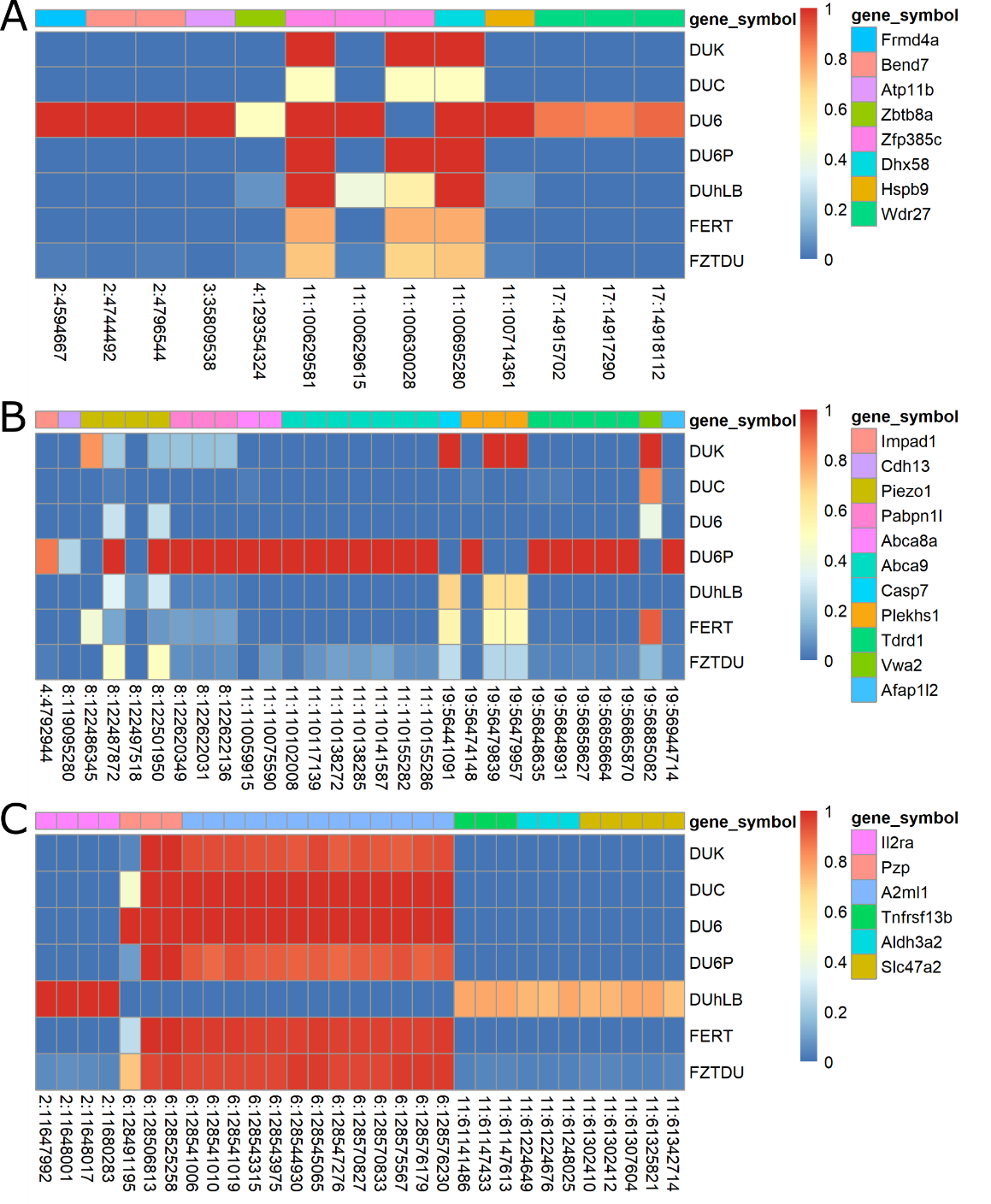
**

**Figure S10. Allele frequency heatmap of non-synonymous mutations in RDD genes.** Allele frequencies of non-synonymous SNPs in genes overlapping regions of distinct genetic differentiation for DU6 (**A**), DU6P (**B**) and DUhLB (**C**). The gradient scale represents the allele frequency from low (blue) to high (red).


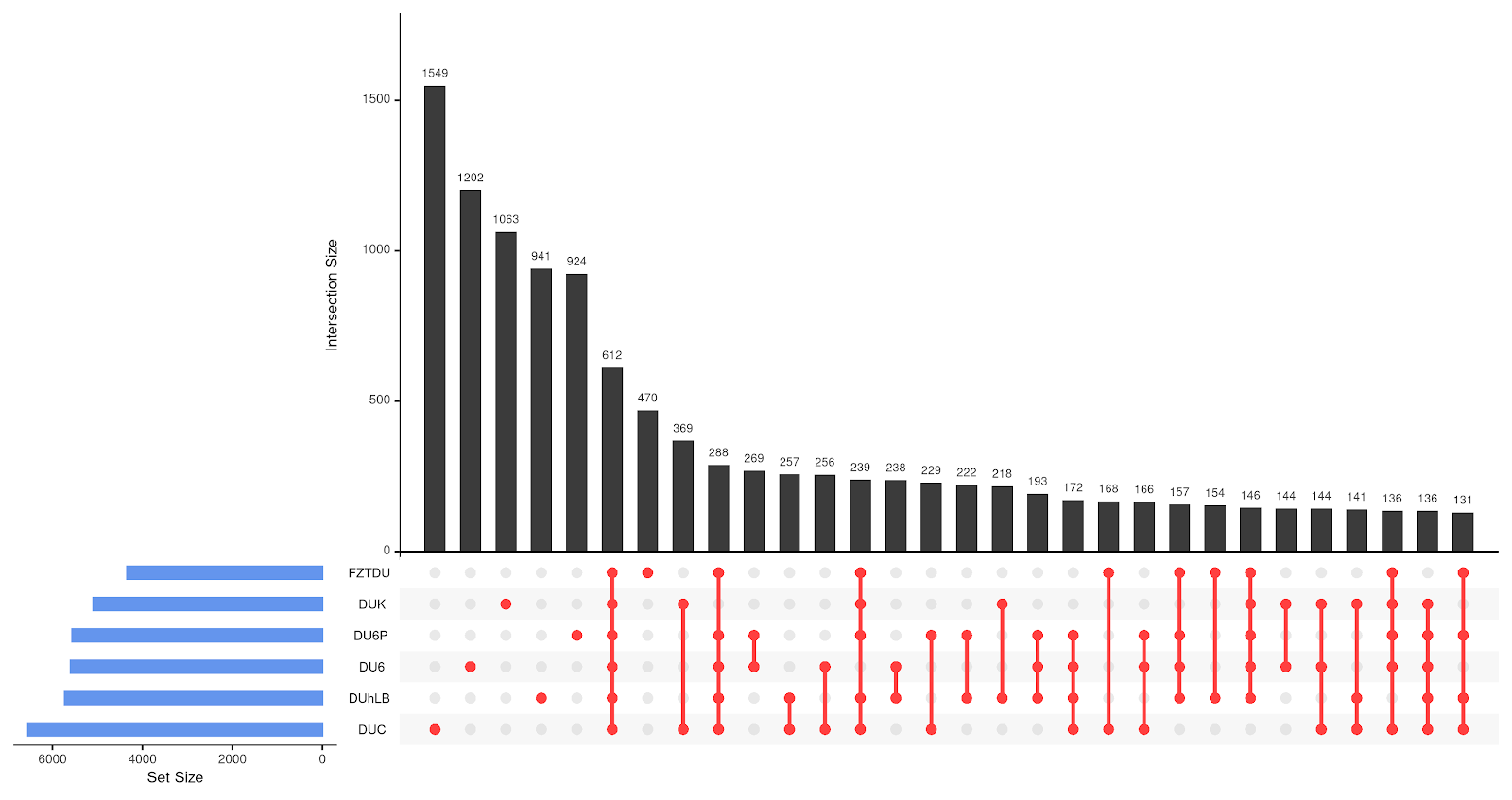


**Figure S11. Shared and line-specific structural variants.** SVs detected in union of high and low coverage sample sets for each mice line.

**
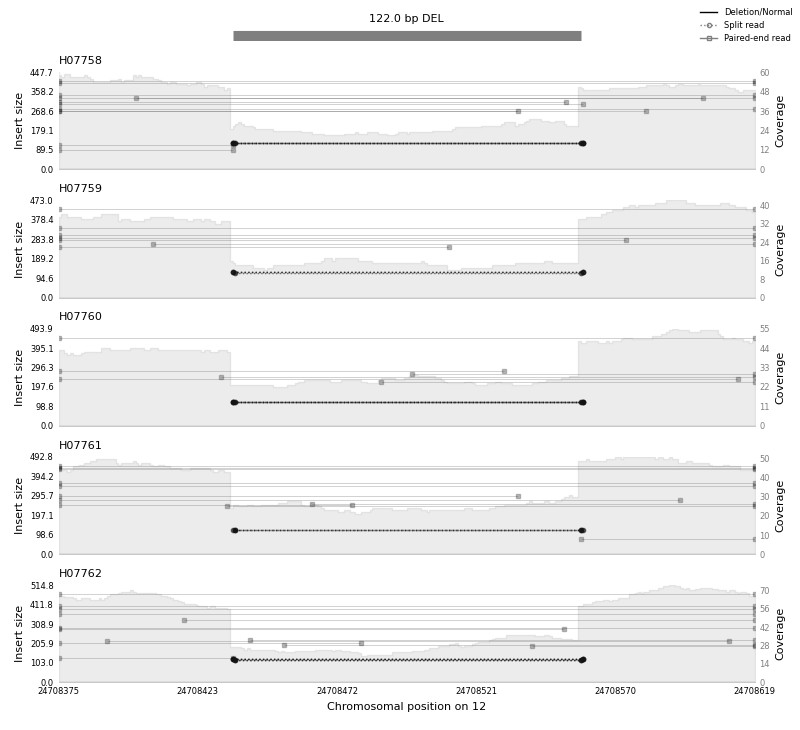
**

**Figure S12. Example of a polymorphic deletion.** SV is shown in 5 out of 10 samples of the high coverage set, indicating the genomic location (x-axis), the insert size (y-axis, left) and coverage (y-axis-right).

**
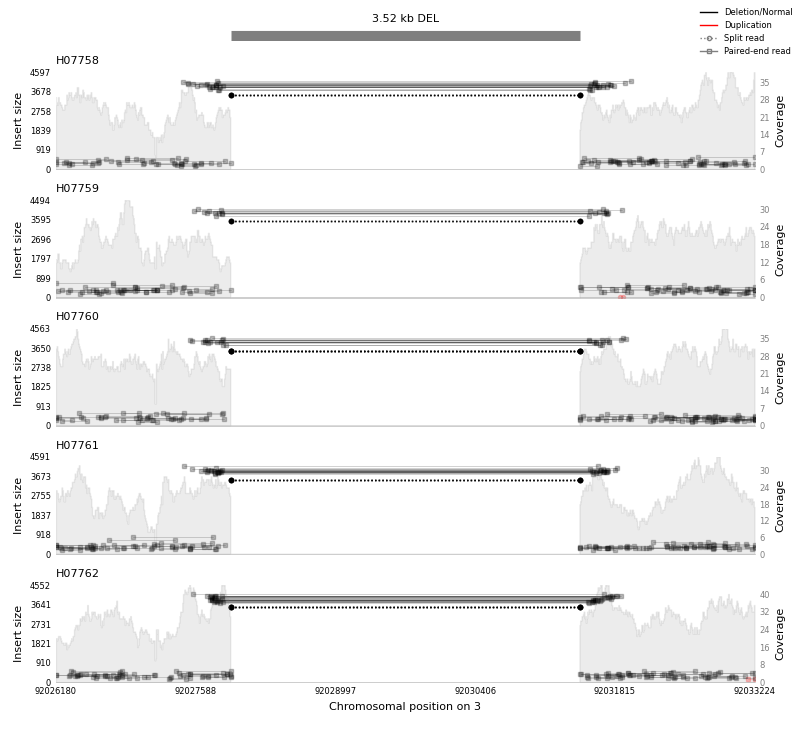
**

**Figure S13. Example of a fixed deletion.** SV is shown in 5 out of 10 samples of the high coverage set, indicating the genomic location (x-axis), the insert size (y-axis, left) and coverage (y-axis-right).

**
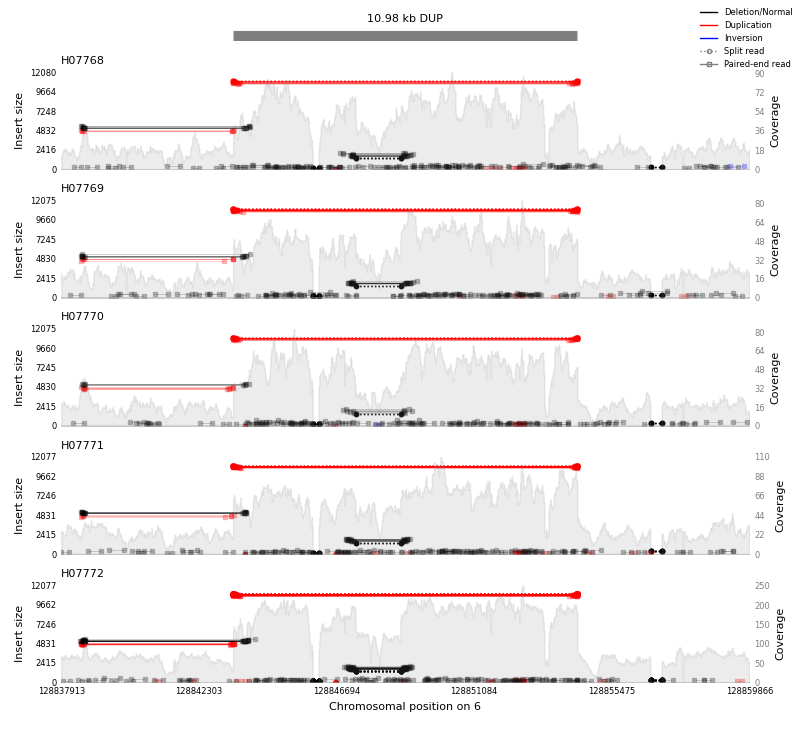
**

**Figure S14. Example of a fixed duplication.** SV is shown in 5 out of 10 samples of the high coverage set, indicating the genomic location (x-axis), the insert size (y-axis, left) and coverage (y-axis-right).

**
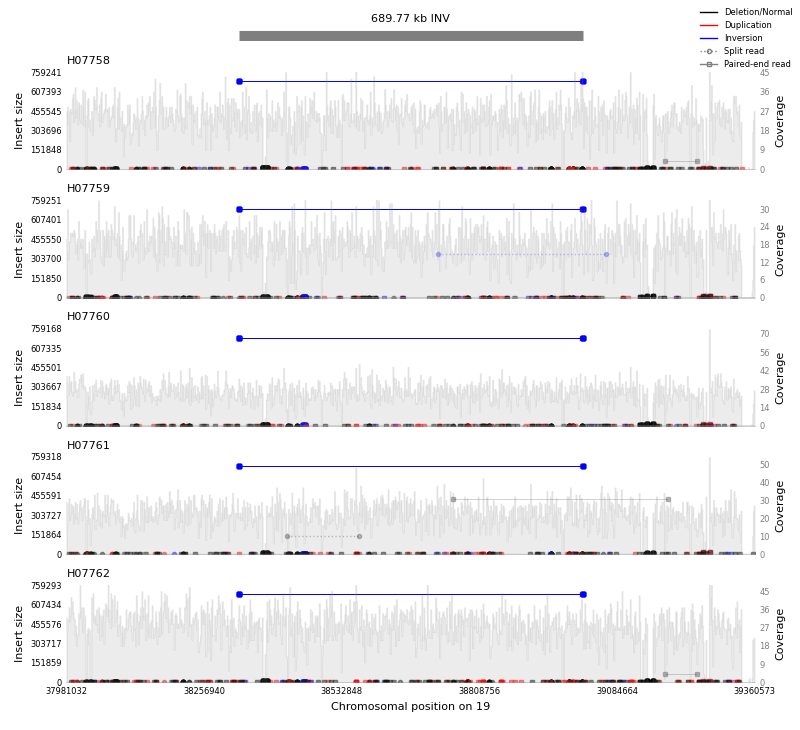
**

**Figure S15. Example of a fixed inversion.** SV is shown in 5 out of 10 samples of the high coverage set, indicating the genomic location (x-axis), the insert size (y-axis, left) and coverage (y-axis-right).
